# PCR-based RFLP and ERIC-PCR patterns of *Helicobacter pylori* strains linked to multidrug resistance in Egypt

**DOI:** 10.1038/s41598-024-72289-z

**Published:** 2024-09-27

**Authors:** Mohammed S. Abdulrahman, Moselhy S. Mansy, Kamel A. Al-Ghreib, Dina Johar, Samy Zaky

**Affiliations:** 1 Microbiology and Immunology Department, Faculty of Pharmacy, Al-Azhar University, Cairo, Egypt; 2 Department of Biochemistry and Nutrition, Faculty of Women for Arts, Sciences and Education, Ain Shams University, Heliopolis, Cairo, Egypt; 3 Hepatogastroenterology and Infectious Diseases Department, Faculty of Medicine, Al-Azhar University, Cairo, Egypt

**Keywords:** *Helicobacter pylori*, Kirby Bauer disc diffusion, Biotyping, Antibiogram typing, PCR-RFLP, ERIC-PCR, MICs, Biochemistry, Biological techniques, Cell biology, Chemical biology, Drug discovery, Microbiology, Molecular biology, Biomarkers, Diseases, Gastroenterology, Medical research, Molecular medicine

## Abstract

*H. pylori* infects approximately 50% of the world’s population that causes chronic gastritis, and may lead to peptic ulcer disease (PUD). *H. pylori*-induced chronic infections are associated with gastric adenocarcinoma and low-grade gastric lymphoma. In Egypt, *H. pylori* strains are widespread and became resistant to antimicrobial agents, thus advanced typing methods are needed to differentiate infectious strains that are resistant to antibiotics, and therefore earlier prognosis and infection control. The main objectives were (i) to determine susceptibility of infectious *H. pylori* strains to some antimicrobial agents that are currently used in eradication therapy in Egypt; (ii) to identify diverse strains commonly detected in the gastrointestinal (GIT) endoscopy units in Egypt through phenotypic and genotypic analyses. In this observational study we isolated 167 isolates from 232 gastric biopsies (antrum and corpus) of patients who were admitted to the upper GIT endoscopy units in five governmental Egyptian hospitals. Antimicrobial susceptibility patterns were investigated using Kirby Bauer disc diffusion and agar dilution Minimum Inhibitory Concentrations (MICs) methods. Phenotypic characterization was based on biotyping and antibiogram typing techniques. Genotypic characterization was carried out using PCR-based Restriction Fragment Length Polymorphism (RFLP) and Enterobacterial Repetitive Intergenic Consensus (ERIC)-PCR analyses. *H. pylori* isolates were highly resistant to diverse antimicrobial agents including Metronidazole, Fluoroquinolones, Macrolides, Amoxycillin, Tetracycline and Gentamicin. Two factors contributed to the increased resistance of *H. pylori* to the conventional therapy seen in Egypt: (i) Metronidazole and Amoxycillin are inexpensive and available drugs being abused by patients; (ii) the regional prescribing practice of Macrolids commonly used to treat upper respiratory and urinary tract infections. Five different biotypes were identified depending on the ability of the isolates to synthesize different enzymes. Nine antibiogram types were identified. PCR-RFLP analysis revealed fifteen different fingerprints while ERIC-PCR revealed 22 fingerprints. Biotyping alone or in combination with antibiogram typing are highly useful molecular tools in the prognosis of strain relatedness. PCR-RFLP and ERIC-PCR acquired good discriminatory power for identifying *H. pylori *infectious sub-types.

## Introduction

*H. pylori* is the most common bacteria infecting about 50% of the World’s population^1^. *H. pylori* inhabit various areas of the stomach, particularly the antrum, causing chronic low-level inflammation of the stomach lining^2^. The infectious strains are strongly linked to the development of duodenal and gastric ulcers, and stomach cancer^3^. Over 80% of patients are asymptomatic^4^. The most important reason for treatment failure appears to be drug resistance^5^. One problem associated with widespread use of antibiotics against *H. pylori* is the generation of resistant strains throughout the world which varies with geographical regions^6^. *H. Pylori* resistant sub-strains is a magnificent health care problem in Egypt. In Egypt, a cohort population-based study was conducted among 169 pregnant women and their offspring, showed 88% prevalence among mothers, 13% among infants 7–9 months of age, and 25% among children 18 months of age. This data indicate that infection with *H. pylori* is common in Egypt and the acquisition of infection occurs at a very young age^7^. There are several methods available to detect *H. pylori* infection including invasive methods that are based on gastric biopsies and endoscopy, followed by microscopy and culture, and non-invasive methods such as serology, urea breath tests, and stool antigen tests^8^. The invasive procedures usually give the most reliable diagnosis, however, these methods are expensive, and not always available^9^.

Successful treatment of *H. pylori* requires combination of two antibiotics plus Proton Pump Inhibitors (PPI)^10^. There are different regimens for treatment including Clarithromycin and Amoxicillin combined with PPI, the Levofloxacin-based triple therapy, the Levofloxacin-based quadruple therapy, and the Rifampicin-based triple therapy. Usually, the course of treatment is 7–10 days, however, it may extend to 14 days^11^. Multiple drug resistance, specially to Clarithromycin causes 70% reduction in the drug’s efficacy, whereas the impact of Metronidazole resistance on the clinical outcome is relatively modest, that is about 25% reduction in efficacy^5^. *H. pylori* resistance to Metronidazole and Clarithromycin has increased worldwide, and multidrug resistant (MDR) strains that are simultaneously resistant to Amoxicillin, Metronidazole and Clarithromycin have been reported^12^.

Bacterial strain typing, including phenotyping and genotyping, are useful molecular techniques for detecting the diversity among strains of the same species^13^. Traditional phenotyping techniques for *H. pylori* included biotyping, antibiogram analysis, lectin typing and serotyping, however, none of those techniques can type all infectious sub-strains^14^. DNA typing methods that target the entire genome are useful for sub-strain differentiation^15^. A wide range of molecular fingerprinting methods is available for epidemiological studies of which amplified 16S rRNA gene restriction analysis and Amplified Fragment Length Polymorphism (AFLP) are the most widely accepted. A number of molecular methods analyzing the genetic relatedness of *H. pylori* isolates have been evaluated, including frequent cutting of Restriction Endonuclease Analysis of chromosomal DNA (REA), macrorestriction of endonuclease analysis using Pulsed-Field Gel Electrophoresis (PFGE), analysis of conserved RNA coding restriction fragments (ribotyping), analyzing the *H. pylori* entire genome, and PCR analysis of i.e. the urease genes^16,17^.

PCR-based fingerprinting methods have been increasingly used in recent years^18^. PCR is an in vitro non-invasive technique for exponentially amplifying a predetermined fragment of DNA. PCR tests for *H. pylori* infection have been based on several genes of *H. pylori* and all have shown good performance. PCR has had high accuracy when applied to formalin-fixed paraffin-embedded specimens^19^.

ERIC sequences are dispersed in the *H. pylori* chromosome at different locations separated by various distances. These widely spread repetitive sequences could be used as primer binding sites and PCR amplification between them might yield divergent fingerprints when separated by agarose gel electrophoresis.

DNA/DNA hybridization and PFGE are gold standard genomic typing methods for bacterial strain discrimination^18,20^. Attempts have been made to distinguish the more pathogenic isolates within *H. pylori* populations using DNA/DNA hybridization in solution and PCR-based DNA fingerprinting techniques such as Randomly Amplified Polymorphic DNA (RAPD)-PCR, PCR-RFLP, and amplified polymorphic DNA using primers with matching consensus-Repetitive Extragenic Palindromic sequences (REP-PCR)^21^.

PCR-based RFLP is a simple, rapid and non-radioactive approach to detect DNA polymorphism. It has been used frequently for typing a variety of bacteria^13^. PCR-based RFLP is a modification of RFLP in which the restriction analysis is performed on PCR amplicons obtained using primers for specific sequences of interest. A DNA banding profile is generated by dissecting the PCR amplicons with restriction enzymes. This method identifies genetic correlation among bacteria and is used in molecular epidemiology study^22^. The limited number of restriction fragments resulting from restriction enzymes digestion of the PCR amplicons can be separated and visualized directly by gel electrophoresis without the need for probe hybridization^23,24^.

This study aimed at isolate and determine the susceptibility profile of infectious *H. pylori* strains, and differentiate pathogenic sub-strains commonly detected in the GIT endoscopy units of outpatient clinics in Egypt, through phenotyping and genotyping variation analyses.

## Results

### Demographic characteristics of patients

*H. pylori* infection was more frequent in male than female patients (56.2% and 43.8%) respectively, and among patients with average age 41–50 years (33.1%). Patients who weighed 77–97 kg (83.5%) were more susceptible to infection than those who weighed 56–76 kg, as shown in Table 1.

**Table 1:** Demographic characteristics of patients with *H. pylori* infection.

	No. of patients	%*		
Sex				
Male	94	56.2		
Female	73	43.8		
Age				
Up to 20 years	9	5.3		
21–30 years	24	14.4		
31–40 years	35	21		
41–50 years	55	33.1		
51–60 years	14	8.3		
61–70 years	22	13.1		

Weight (kg)	Occurrence		*H. pylori* +ve isolates	
	No	%*	No	%**
35–55	43	18.5	30	69.7
56–76	55	23.7	36	65.4
77–97	71	30.6	59	83.1
98–120	63	27.2	42	66.6

Smoking habit	Patients		*H. pylori* +ve isolates	
	No	(%)	No	%
Smokers	62/232	26.7	44/167	70.9
Non-smoker	133/232	57.3	104/167	78.2
Ex-smokers	37/232	16.0	19/167	51.3

Coffee drink	*H. pylori* −ve		*H. pylori* +ve	
	No	%	No	%
No	39	35.8	70	64.2
Yes	26	21.2	97	78.8
1–5 cups/day	92	74.8	71	77.1
> 5 cups/day	31	25.2	26	83.8

*Numbers were calculated as a percentage of the total number of patients.

**Numbers in the column were calculated as a percentage of the total number of isolates.

Isolates were Gram negative motile curved *bacilli*, with a typical biochemical profile of *H. pylori* as shown in Table 2. The rate of isolation was shown in Supplementary Table S.T.1. The highest rate of isolation was seen in selective Brain Heart Infusion (BHI) blood agar (67.0%).

**Table 2:** Biochemical reactions for identification of *H. pylori* isolates.

Biochemical reaction	*H. pylori*	Biochemical reaction	*H. pylori*
Urease	+ve	Growth with 3.5% NaCl	−ve
Catalase	+ve	Growth on peptone-starch dextrose (PSD) agar	+ve
Oxidase	+ve	Growth on 1% glycine	−ve
H2S production	−ve	Cephalothin resistant	−ve
Nitrate reduction	−ve	Nalidixic acid resistant	+ve
Growth at 42 °C	−ve	Hippurate hydrolysis	−ve
Growth at 25 °C	−ve	Indole formation	−ve

Supplementary Table S.T.2 showed that the most predominant complaint of patients with *H. pylori* infection was epigastric pain in 133 patients (79.6%) followed by vomiting and distention in (32.3%) and (22.1%) respectively. The upper GIT endoscopy revealed the most prevalent diagnosis associated with *H. pylori* infection was gastritis (88.8%), of which (31.0%) tested positive for *H. pylori*. Gastric ulcer (GU), duodenitis, patients with normal gastric mucosa, duodenal ulcer (DU), and gastroesophageal reflux disease (GERD) had less prevalence of positive *H. pylori* isolates 74.4%, 69.4%, 63.3%, 57.9% and 50.0% respectively.

### Antimicrobial susceptibility tests

Table 3 shows frequency of antimicrobial susceptibility among 167 isolates by standard disc diffusion method. The highest susceptibility (88.6%) was agonist Levofloxacin, followed by Erythromycin (84.4%). In contrast, Metronidazole showed the highest resistance pattern (83.2%), followed by Amoxicillin (67.6%).

**Table 3:** Frequency of antimicrobial susceptibility of *H. pylori* isolates using disc diffusion method.

Antimicrobial agent	Abbreviation	*H. pylori* (N = 167)		
		Sensitive isolates (%*)	Intermediate sensitive isolates (%*)	Resistant isolates (%*)
Metronidazole	MTZ	10 (6.0)	18 (10.8)	139 (83.2)
Amoxicillin	AM	54 (32.4)	–	113 (67.6)
Clarithromycin	CLA	119 (71.2)	–	48 (28.7)
Tetracycline	TE	88 (52.7)	-	79 (47.3)
Erythromycin	E	141 (84.4)	–	26 (15.6)
Levofloxacin	LEV	148 (88.6)	–	19 (11.4)
Ciprofloxacin	CIP	132 (79.0)	–	35 (21.0)
Furazolidone	FX	101 (60.4)	27 (16.1)	39 (23.4)
Gentamicin	GM	115 (68.9)	–	52 (31.1)
Rifampicin	RD	109 (65.3)	–	58 (34.7)

*Numbers were calculated as a percentage of the total number of isolates.

Table 4 shows antimicrobial susceptibility tests of 167 *H. pylori* isolates using MICs by agar dilution method. The highest susceptibility rate was 141 isolates agonist Levofloxacin (84.5%), followed by 138 (82.6%) to Erythromycin. Metronidazole showed the highest resistance pattern 153 (91.7%), followed by Amoxicillin 119 (71.2%). The two methods validated each other. A high throughput profiling of the 167 isolates susceptibilities by the two methods is shown in Appendix A.

**Table 4:** Prevalence of antibiotic-resistant *H. pylori* isolates using MICs method.

Antimicrobial agents	Abbreviation	*H. pylori* (N = 167)		
		Sensitive Isolates (%*)	Intermediate sensitive isolates (%*)	Resistant isolates (%*)
Metronidazole	MTZ	14 (8.3)	–	153 (91.7)
Amoxicillin	AM	48 (28.8)	–	119 (71.2)
Clarithromycin	CLA	93 (55.7)	21 (12.7)	53 (31.7)
Tetracycline	TE	96 (57.5)	–	71 (42.5)
Erythromycin	E	138 (82.6)	–	29 (17.4)
Levofloxacin	LEV	141 (84.5)	–	26 (15.5)
Ciprofloxacin	CIP	121 (72.4)	–	46 (27.6)
Furazolidone	FX	119 (71.2)	–	48 (28.8)
Gentamicin	GM	110 (65.9)	–	57 (34.1)
Rifampicin	RD	125 (74.9)	–	42 (25.1)

*Numbers were calculated as a percentage of the total number of isolates.

### Phenotypic characterization of *H. pylori*

#### Enzyme profiles of biotypes

Enzyme profiles of fifty *H. pylori* strains were characterized according to Matsumoto scheme (1996)^25^. All strains assessed were urease-, catalase-, and oxidase positive. All strains assessed exhibited acid phosphatase, leucine arylamidase and alkaline phosphatase activity and differential activity for esterase, esterase lipase and naphthol-AS-(β1-phosphohydrolase). Alkaline phosphatase, acid phosphatase (100% for each) and leucine arylamidase (98%) presented the highest percentage of positive reactions, whereas, esterase, esterase lipase and naphthol-AS-β1-phosphohydrolase presented lower (18%), (16%) and (20%) positive reactions respectively. The API ZYM cut off table is shown in (Supplementary Table S.T.3). Out of the 50 strains, 6 (12%) were biotype Ia, 1 (2%) was biotype Ib, 27 (54%) were biotype IIa, 2 (4%) were biotype IIb, 10 (20%) were biotype III and 4 (8%) were unidentified, Table 5.

**Table 5:** Biotyping scheme of *H. pylori* strains.

Enzymes	Biotypes					
	Ia	Ib	IIa	IIb	III	IV
Alkaline phosphatase	+	+	+	+	+	+
Esterase (C4)	+	+	−	−	−	−
Esterase lipase (C8)	+	+	−	−	−	−
Leucine arylamidase	+	−	+	−	+	−
Acid phosphatase	+	+	+	+	+	+
Naphthol-AS-β1-phosphohydrolase	+	+	+	+	−	−
Total	6	1	27	2	10	4
%*	12	2	54	4	20	8

*Numbers were calculated as a percentage of the total number of isolates.

#### Antibiogram typing of *H. pylori* strains

The antibiogram typing revealed nine antibiotypes. These antibiotypes were arbitrarily given large case alphabets as A1 to A9 as per the scheme of Ndip et al.^26^. Such pattern is shown in Table 6.

**Table 6:** Antibiogram patterns of *H. pylori* isolates (n = 50).

Antibiotype	Pattern	NO. (%)*
A1	Resistant to all antimicrobial agents	3 (6.0)
A2	Resistant to Clarithromycin only	6 (12.0)
A3	Resistant to Amoxicillin only	4 (8.0)
A4	Resistant to Metronidazole and Amoxicillin	11 (22.0)
A5	Resistant to Metronidazole and Clarithromycin	9 (18.0)
A6	Resistant to Metronidazole, Levofloxacin and Furazolidone	6 (12.0)
A7	Resistant to Levofloxacin, Clarithromycin and Gentamycin	5 (10.0)
A8	Resistant Metronidazole, Tetracycline, Clarithromycin and Amoxicillin	4 (8.0)
A9	Resistant to Amoxicillin, Clarithromycin, Erythromycin, Ciprofloxacin and Rifampicin	2 (4.0)

*Percentage to total number of isolates.

#### ERIC-PCR analysis

**Fig. 1:**
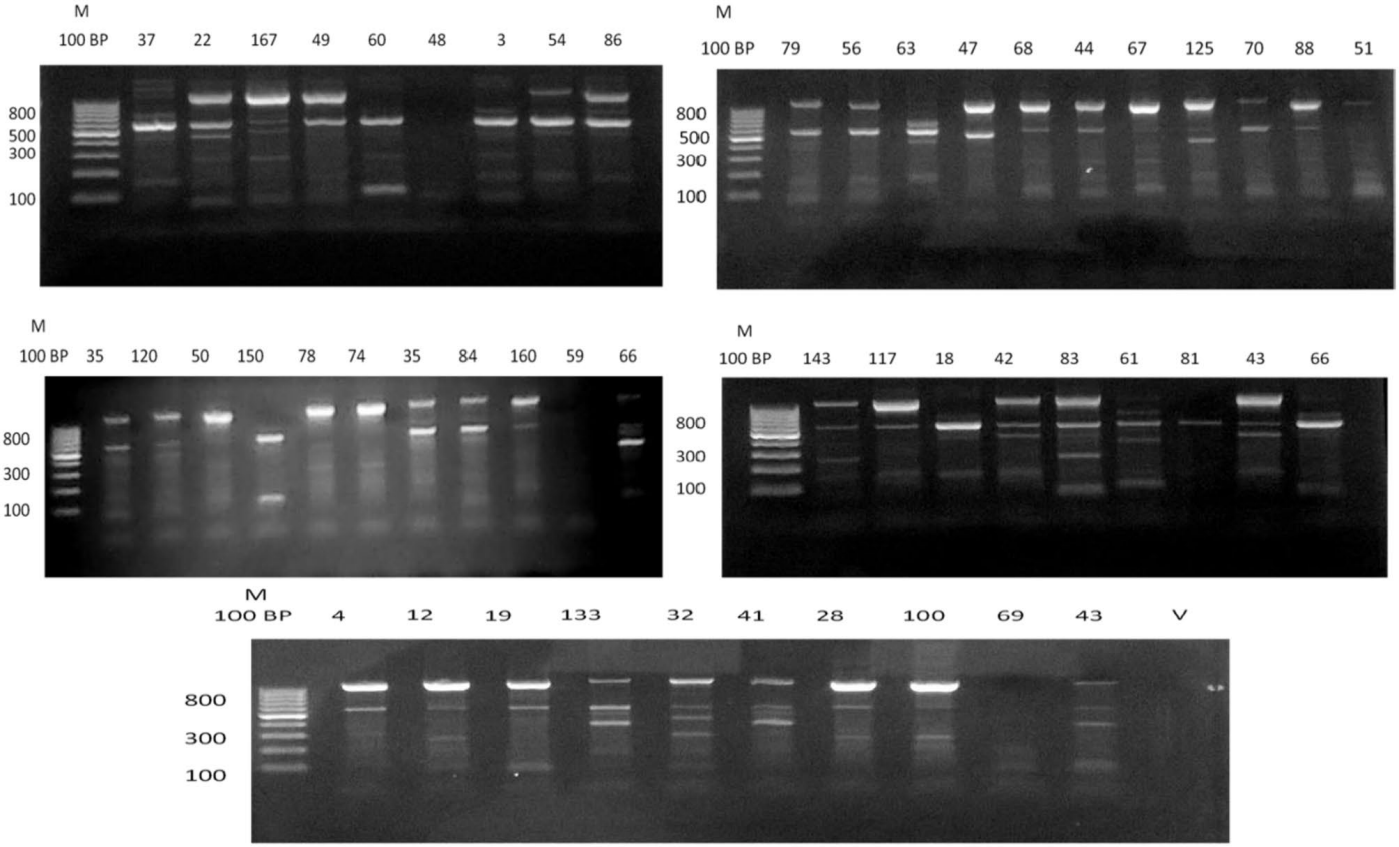
DNA fingerprints generated by ERIC-PCR analysis of *H. pylori* isolated products on 2% agarose gel: M is 100 bp DNA ladder, (V) is a negative control (n = 50).

Genotyping analysis of all strains was validated by the ERIC-PCR method and yielded significant PCR products. All *H. pylori* isolates gave 126 bp, typical for ERIC profiles, and 9 bands with different sizes resulted in 22 genotypes. Those genotypes were arbitrarily given large case alphabets as G1 to G22. The most predominant genotype was G2 that was detected in 8 (16.0%) isolates, followed by G4 that was detected in 7 (14.0%) isolates, as illustrated in (Supplementary Table S.T.4 and Fig. 1).

### PCR amplification of *H. pylori ureC* gene

#### PCR-RFLP analysis

**Fig. 2:**
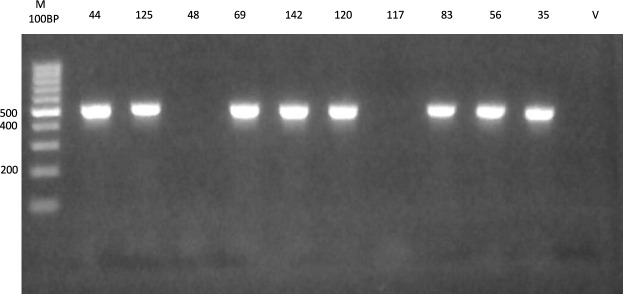
PCR amplification products of *H-pylori ureC* gene yielded 500 bp bands. V, negative control and M, 100 bp molecular weight marker.

**Fig. 3:**
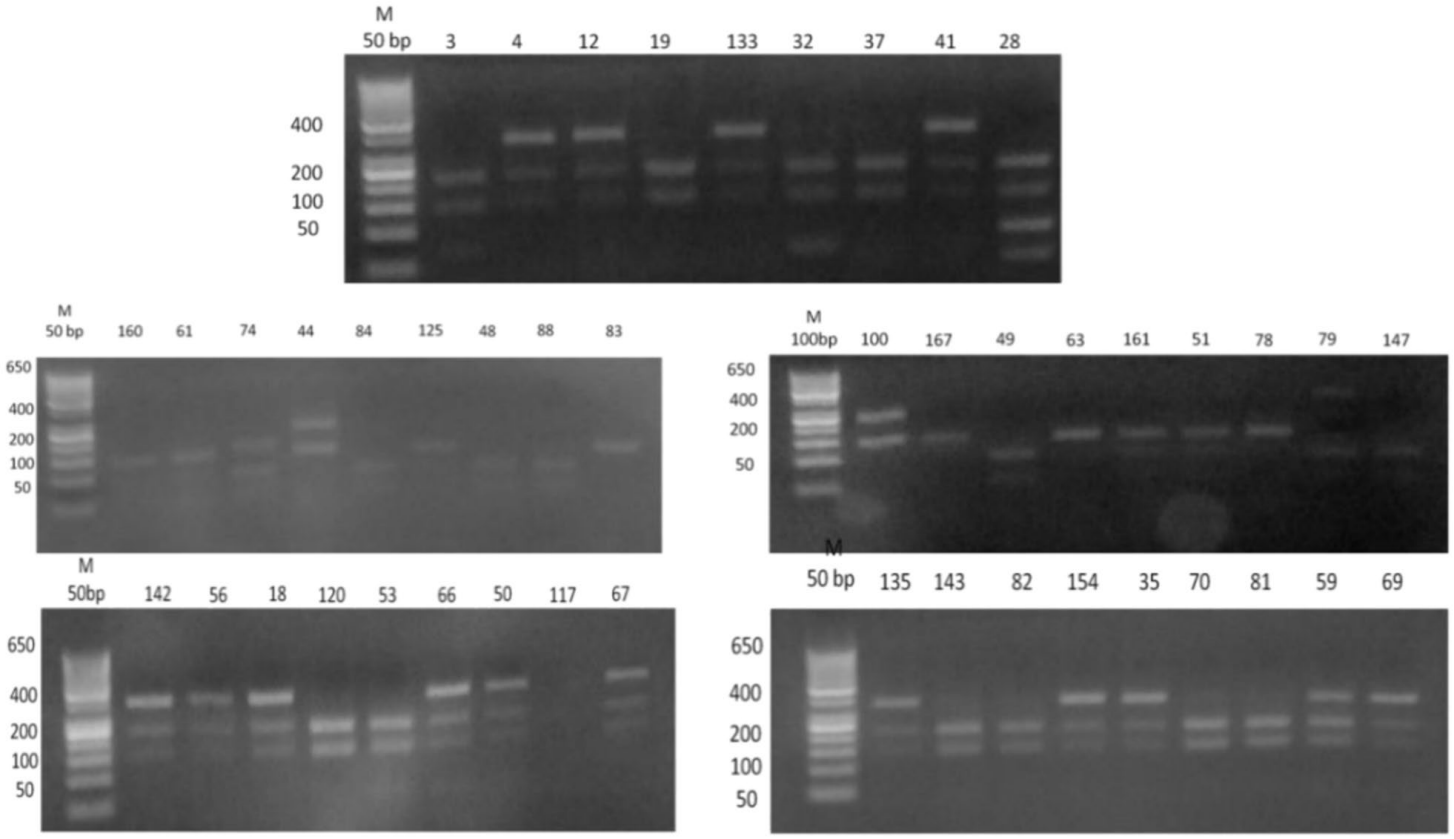
PCR-based RFLP patterns of *H. pylori* isolates digested with *Mbo*I restriction enzyme (n = 44).

PCR amplification of urease positive *H. pylori* DNA using *ureC* gene yielded suspected 500 bp product sizes in 44 isolate (88.0%), while 6 isolates were negative (12.0%), Fig. 2. The products of the 44 PCR positive specimens were digested with *Mbo*I restriction enzyme that produced 11 bands with different sizes resulted in 15 genotypes detectable by agarose gel electrophoresis. Those genotypes were arbitrarily given large case alphabets as M1 to M15. The most predominant genotype was M4 that was found in 8 (18.1%) of isolates, followed by M9 and M2 that were detected in 7 (15.9%) and 6 (13.6%) isolates respectively as shown in (Supplementary Table S.T.5, and Fig. 3).

#### The Simpson index of diversity (D)

**Fig. 4:**
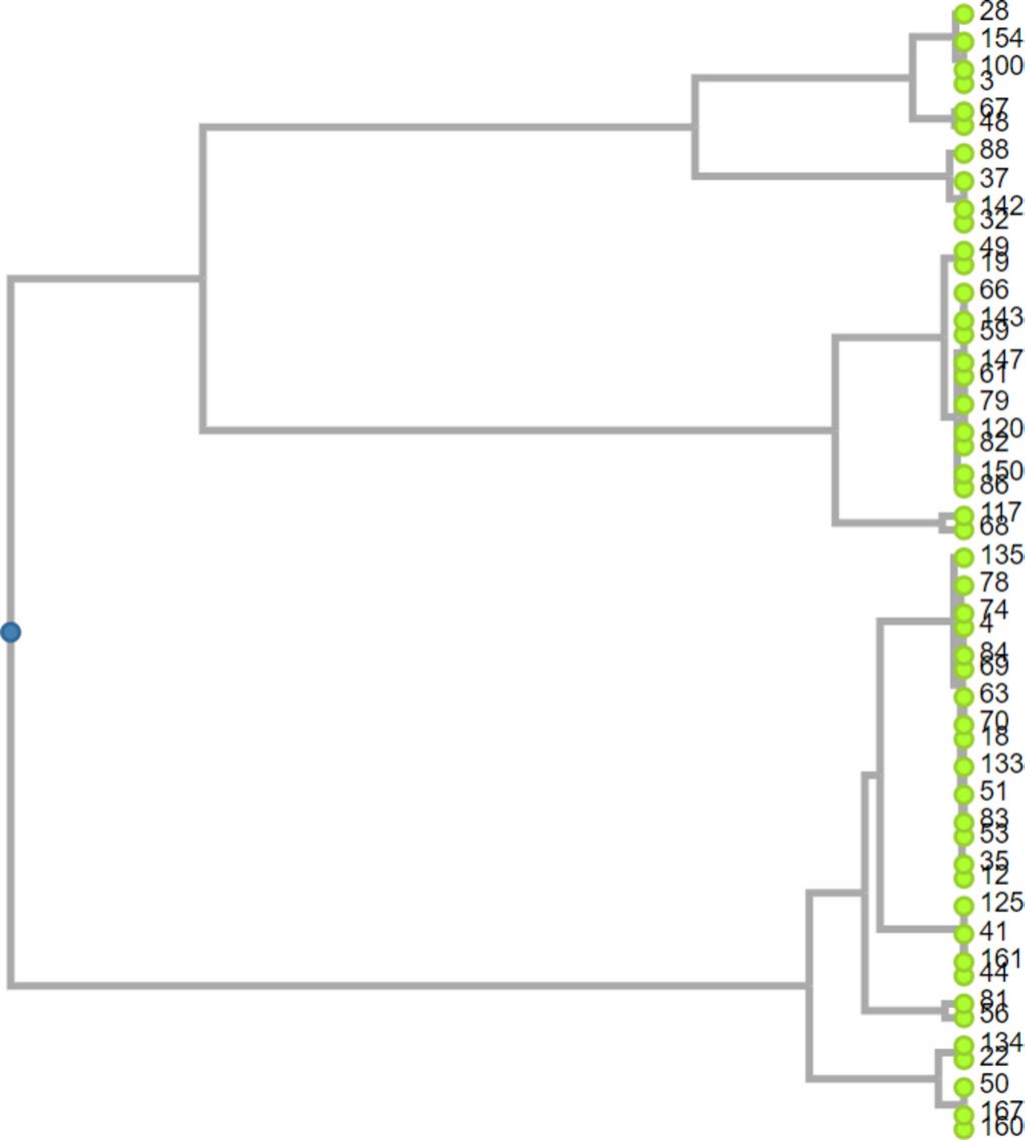
A dendrogram elucidating *H*. *pylori* isolates relatedness.

Biotyping, antibiogram typing, PCR-RFLP and ERIC-PCR typing methods scored 0.61, 0.88, 0.91 and 0.94 respectively. The highest discriminatory power was corresponding to ERIC-PCR as shown in Table 7. Genotypes, antibiogram types and biotypes for each isolate involved in this study and their correlation with clinical disorder was summarized in Table 8. A detailed calculation of the D index is shown in Appendix B. A dendrogram elucidating *H. pylori* isolates relatedness is shown in Fig. 4.

**Table 7:** Simpsons' index of diversity for *H. pylori* isolates.

Method	No. of different types	No. of isolates belonging to most numerous types	Simpsons' index of diversity
ERIC-PCR	22	6	0.94
PCR-RFLP	15	7	0.91
Antibiogram typing	9	11	0.88
Biotyping	5	27	0.61

**Table 8:** Summary of biotyping, antibiogram typing and genotyping of different *H. pylori *isolates and their correlation with clinical disorders.

No.	Isolates	Phenotyping		Genotyping		Clinical outcome
		Biotyping	Antibiotyping	ERIC-PCR	PCR-RFLP	
1	3	Ia	A8	G1	M1	Gastritis
2	4	Iia	A7	G2	M2	Gastritis
3	12	Iia	A9	G6	M3	GU
4	18	Iia	A3	G4	M2	Gastritis
5	19	Ia	A1	G14	M4	DU
6	22	Iia	A4	G2	–	Deodenitis
7	28	Ib	A4	G8	M9	Gastritis
8	32	Iia	A4	G9	M12	DU
9	35	Iia	A9	G8	M2	GU
10	37	Iia	A7	G2	M12	Gastritis
11	41	III	A4	G6	M9	Gastritis
12	44	III	A2	G2	M4	Gastritis
13	48	Ia	A8	G21	M10	GU
14	49	Ia	A6	G12	M2	GU
15	50	III	A3	G10	M3	Gastritis
16	51	Iia	A3	G3	M3	GERD
17	53	Iia	A4	G2	M2	Deodenitis
18	56	Iia	A3	–	M4	Gastritis
19	59	Iia	A5	G10	M7	GU
20	61	Iia	A2	G11	M2	Normal
21	63	Iia	A5	G7	M4	GERD
22	66	Iia	A5	G11	M9	Gastritis
23	67	Iia	A1	G13	M12	DU
24	68	–	A6	G2	–	Gastritis
25	69	Iia	A5	G2	M4	GU
26	70	Iia	A7	G7	M5	Normal
27	74	Iia	A7	G2	M4	Gastritis
28	78	Iia	A4	G5	M9	Deodenitis
29	79	Iia	A7	G11	M5	GERD
30	81	III	A6	G4	M13	Normal
31	82	Iia	A4	G12	M4	GU
32	83	Iia	A4	G4	M4	Gastritis
33	84	Iia	A5	G2	M6	GU
34	86	Iia	A2	G12	–	Gastritis
35	88	Iia	A6	G9	M14	Deodenitis
36	100	–	A2	G2	M6	Gastritis
37	117	Ia	A4	G5	–	DU
38	120	Iia	A5	G14	M5	Deodenitis
39	125	III	A8	G2	M9	Normal
40	133	Iia	A4	G4	M6	GU
41	134	Iib	A6	G4	–	Gastritis
42	135	III	A7	G21	M11	GERD
43	142	Iia	A7	G9	M14	DU
44	143	III	A6	G12	M8	Gastritis
45	147	Iia	A2	G16	M2	Deodenitis
46	150	Iib	A5	G17	–	Gastritis
47	154	–	A5	G4	M9	Gastritis
48	160	III	A4	G18	M6	Gastritis
49	161	III	A4	G4	M4	GU
50	167	III	A5	G18	M9	GERD

*A* antibiotype, *G* genotype using ERIC-PCR, *M* genotype using RFLP-PCR.

## Discussion

In the present study, a total of 167 *H. pylori* isolates were collected from 232 antral and corpus biopsies of outpatients at three different upper GIT units in Egypt. The isolation rate of *H. pylori* (72%) was higher comparing to other studies. For example, Abu-Sbeih et al.^8^ reported *H. pylori* isolation at a rate of 53.3%, Al-Sulami et al. reported 67.6%^27^, and Amer et al., 91%^28^. This variation was attributed to the versatile prevalence of *H. pylori* in different environments, socioeconomic states, hygienic conditions, and sensitivity of diagnostic methods. For example, globally, the prevalence of *H. pylori* infection in developing countries is markedly higher than that in developed countries^29^, and relies on the rate of acquisition throughout childhood^30^, and the difference in socioeconomic status rather than ethnicity^3^. Approximately 30% of patients with dyspepsia in North America are infected with *H. pylori*^31^*,* compared to 80–90% in the developing nations^32^. The variations in the prevalence of infection between and among populations may point to the fact that parameters such as age, cultural background, genetic predisposition, socio-economic status and environmental factors all play a role in the acquisition and transmission of infection^33,34^.

A number of authors have emphasized the role of other factors such as smoking, alcohol consumption, occupational exposure, waterborne exposures, hygienic practices, density/crowding, social factors and family history of gastric disease. Most Africans might be at risk of becoming infected with the organism due to the primitive water sources that are the main water sources in some communities^34^. Food-borne transmission and unclean hands have also been involved in transmission^35^. Others observed a significant relationship between *H. pylori* infection associated with gastric malignancy and blood group type A^36^.

In this study, *H. pylori* infection did not statistically differ among smokers, non-smokers and ex-smokers. Patients who usually drink coffee had higher frequency of infection than those who did not usually drink (78.8%) and (64.2%) respectively, *p* = 0.0098. Education level significantly impacted the rate of infection. The highest infection rate was detected within intermediate school group, whereas the lowest rate was within university patients, *p* = 0.0046.

Based on the simplified phenotypic identification tests reported^27,37^, the isolates obtained were identified as *H. pylori,* since they were microaerophilic Gram negative curved rods. The cultured bacteria were motile with sluggish movement, showed positive reactions for oxidase, catalase and urease. The bacteria were confirmed to have negative reaction in hippurate hydrolysis, nitrate reduction, H2S production, indole formation, growth at 42 °C, 25 °C, in 1% glycine and 3.5% NaCl. Those findings were consistent with others^8,38^.

Emergence of multiple drug-resistant strains of *H. pylori* is a problem for treatment guidelines, as they reduce the efficacy of current regimens for the eradication of *H. pylori* that is commonly associated with prior antibiotic use. Most patients are prescribed initial *H. pylori* eradication treatment without culture and antibiotic susceptibility testing as current guidance recommend a ‘‘test-and-treat’’ strategy based on non-invasive diagnostic methods. This study reports highly resistant patterns of *H. pylori* strains in Egypt, consistent with other reports^39,40^. The resistance rate to Metronidazole detected by MICs was 91.7%, and 83.2% by standard disc diffusion method. This data is consistent with reports from developing countries that described high resistance to Metronidazole as (66.2–100%)^41^. The use or abuse of this inexpensive drug may contribute to the increased Metronidazole resistance seen in developing countries, including Egypt. The controversy about treatment failure with Metronidazole was previously attributed to the methodological problems in determination of Metronidazole resistance of *H. pylori*^42^, and patient’s compliance with the prescribed regimen. Noncompliance is occurrence of side effects^43^. Further, nonconvenience plays a role: i.e., patients receiving a 2–3-times-a-day regimen were significantly more compliant than those receiving a 4-times-a-day regimen^44^. Irrespective of patient compliance, eradication treatment appears to be more effective in patients with peptic ulcer disease than without it^45^.

The present study revealed a high resistance rate to Amoxicillin 67.6% by standard disc diffusion method, and 71.2% by agar dilution method. This data agrees with Fathi et al.^39^, that showed resistance rate to Amoxicillin of 87.5% using MICs method, and with Hussein^46^, that showed resistance rate of 60% using disc diffusion technique. Our data support the findings of Aboderin et al.^47^, who reported 100% resistance in all the examined 32 isolates. This wide variation in Amoxicillin resistance rates reported from different countries could be attributed to the regional prescribing practice of the drug.

Clarithromycin is recommended as the first line triple therapies against *H. pylori*. The increase of Clarithromycin resistance has been found to be one of the main risk factors for treatment failure in most countries^48^. This study revealed a resistance rate of 28.7% to Clarithromycin by the disc diffusion method, and 31.7% to Clarithromycin by agar dilution method. This finding agrees with Ilie et al.^49^ who reported a resistance rate of 32% to Clarithromycin for their tested isolates, and contradicts with Fathi et al.^39^ who reported 100% resistant rate to Clarithromycin by disc diffusion method; and 50% (8 out of 16 isolates) to Clarithromycin by MICs method. The high rate of Clarithromycin resistance reported by the last study is likely a consequence of an overuse of Macrolides for the treatment of upper respiratory tract infections, and cross resistance between Erythromycin and Clarithromycin.

In this study we detected 71 resistant strains to Tetracyclines, out of the 167 tested isolates (47.3%) by standard disc diffusion method, compared to 111 out of 167 (42.5%) by agar dilution method. Our data contradict the findings of Boyanova et al.^50^, and Saeed et al.^6^, which revealed relatively low resistance to Tetracycline (4% and 16.6%) respectively. In our study, the increased resistance of *H. pylori* against Tetracycline can be attributed to the low price and wide availability of the drug without prescription commonly practiced in Egypt.

Ciprofloxacin belongs to the Fluoroquinolone group of antibiotics that are generally used as part of regimens for *H. pylori* infections when first and second line therapies fail^51^. Resistance to Fluoroquinolones is generally very low (< 10%) worldwide^50^. In this study, we report low resistance to Ciprofloxacin using the disc diffusion method (35 out of 167 isolates) 21.0%, versus 27.6% that was reported by agar dilution method. This increase in resistance to Ciprofloxacin could be attributed to the high frequency of its prescription in respiratory and urinary tract infections.

Clarithromycin is key antibiotic in triple therapies for *H. pylori* infections. Resistance to Clarithromycin is responsible for low efficacy of triple therapy. As an alternative to Clarithromycin, the new second-line treatment regimens that contain Levofloxacin have been disappointing due to the emerging Levofloxacin resistance^52^. As to the resistance rate of Levofloxacin, the current study revealed 15.5% (26 out of 167 isolates) by agar dilution method, versus 11.4% (19 out of 167 isolates) by standard disc diffusion method. The finding of our study is consistent with Loffeld and Werdmuller^53^ who reported (14.1%) resistance rate, and contradicts Saeed et al.^6^, who did not find resistance of *H. pylori* against Levofloxacin. Triple therapy combining a PPI, Levofloxacin, and Amoxicillin has been proposed as an alternative to the standard rescue therapy, this salvage regimen can achieve a higher eradication rate than bismuth-containing quadruple therapy in some regions and has fewer adverse effects. Levofloxacin-based triple therapy consisting of Levofloxacin (500 mg, once daily), Amoxicillin (1 g, twice daily), and a PPI (standard dose, twice daily) represents an encouraging strategy for second-line therapy^54^.

Recently, sequential treatment consisting of five days of a PPI plus Amoxicillin, followed by five additional days of a PPI plus Clarithromycin and Tinidazole, has been shown to be better than the combination of a PPI plus Amoxicillin and Clarithromycin for 7 days^55^. Novel first-line anti-*H. pylori* therapies include sequential therapy, concomitant quadruple therapy, hybrid (dual-concomitant) therapy, and bismuth-containing quadruple therapy. Moreover, in cases of patients for whom there was a failure of the standard triple therapy, a bismuth-containing quadruple therapy comprising a PPI, bismuth, Tetracycline, and Metronidazole can be employed as rescue treatment^56^.

Another possible treatment is the use of Furazolidone, with a PPI and Clarithromycin or Tetracycline, however, due to the possible genotoxic and carcinogenetic effects, Furazolidone use is not approved in developed countries^57^. In the present study, 23.4% (39 out of 167 isolates) was resistant to Furazolidone using standard disc diffusion method, versus 28.8% (48 out of 167 isolate) by the MICs method. Our study resistance rates were higher than 5.0% by Amer et al.^28^ and 6.72% by Ghotaslu et al.^58^, and contradict the Brazilian study^59^ which reported 100% of patients were sensitive to Furazolidone.

In this study, the prevalence of Erythromycin resistance was 15.6% (26 out of 167 isolates) by disc diffusion method, similarly to (13.8%) that was reported by Pandya et al.^60^. Higher rates 44.5% and 26.0% were reported by Tanih et al.^35^ and Loffeld and Werdmuller^53^ respectively.

Rifampicin has potential activity against *H. pylori* because of its high sensitivity in vitro, as it does not share resistance to either Clarithromycin or Amoxicillin^61^. We report 42 resistant isolates to Rifampicin (25.1%) by MICs agar dilution method. Our data agrees with Dadashzadeh et al. who found 26.9% of *H. pylori* isolates resistant to Rifampicin^62^.

In the current study, Gentamicin resistant strains were 31.2% (52 out of 167 isolates). Our study agrees with data from South-Africa that showed higher resistance (27.5%) to Gentamicin corresponding to 55 out of 200 isolates^35^. In contrast to data from China that revealed very low resistance rate in 0.1% of *H. pylori* positive patients^63^.

### The preformed enzyme profiles

The performed enzyme profiles of 50 clinical *H. pylori* strains were assessed. According to the Matsumoto et al. biotyping scheme^25^, this study reported 50 strains; 12% (6/50) were biotype Ia, 2% (1/50) were biotype Ib, 54% (27/50) were biotype IIa, 4% (2/50) were biotype IIb, 20% (10/50) were biotype III and 8% (4/50) unidentified. Our data agreed with Uyub and Azlan (2000) where 46.2% (24 out of 52 isolates) were biotype IIa, followed by biotypes Ia and IIb (38.5% and 3.8%) respectively^64^. Our data contradict a report by Matsumoto et al. that the most abundant strain was biotype IIb (49.5%), followed by biotype Ib (37%), while the remaining 13.5% were included among the other biotypes^25^.

Resistance of *H. pylori* to the available antibiotic treatments is a magnificent health problem^65^. The current study revealed nine antibiogram types (antibiotypes), that were identified among the 50 investigated *H. pylori* isolates, and were designated as A1 to A9. The antibiotype A4 was the most predominant, identified in (20.0%) of isolates, followed by antibiotype A4 in 18.0% isolates. Isolates related to antibiogram type A4 were sensitive to all classes of antimicrobial agents examined except Metronidazole and Amoxicillin, while antibiogram type A5 included *H. pylori* sensitive to all antimicrobial classes used except Metronidazole and Clarithromycin. In summary, multidrug resistance in our study can be explained by the frequent usage of antimicrobials and the indiscriminatory use of those antibiotics to treat different other infectious conditions.

### ERIC sequence-based genotyping

Molecular methods like PCR have been used extensively for the identification of *H. pylori* from gastric biopsy specimens^66^. PCR yields information on the presence of potential virulence markers in the strain, which might have implications for the development of severe disease or efficacy of eradication^67^. Different primers have been utilized and some have been developed into commercial kits. Different loci have been used as the target for the amplification: 16S rRNA; A-B- and C-urease; flaA; CagA; vacA and Heat-Shock Protein (HSP). Real time results can be obtained using light cycle technology^67^.

ERIC sequence-based genotyping was identified as a novel method for phylogenetic analysis of *H. pylori*. Those sequences are examples of bacterial interspersed mosaic elements that appear to be transcribed, although they may exist in the intergenic regions or in the 3′ or 5′ untranslated regions of the protein coding regions. The presence of intergenic regions in the bacterial chromosome helps in controlling potential signals for the processes of transcription and translation^68^. In this study, ERIC-PCR typing revealed 22 genotypes of 50 *H. pylori* isolates. This was consistent with Hussein who reported 26 genotypes among 61 clinical isolates of *H. pylori* using the same technique^68^. Polymorphic ERIC patterns indicated that the ERIC sequences were dispersed in the *H. pylori* chromosome at different locations separated by various distances.

### PCR-RFLP analysis of the *ureC* gene

Several studies have confirmed that PCR-RFLP analysis of the *ureC* gene can discriminate *H. pylori* isolates in clinical specimens. Using restriction endonucleases, Moore et al. analyzed the 1.1 kb portion of the *ureC* gene that was amplified by the PCR-RFLP of 21 *H. pylori* isolates, similarly done in our study^69^.

### Genetic variability of *H*. *pylori* isolates

This study analyzed the genetic variability of *H. pylori* isolates by the enzyme *MboI*. Digestion of a 417-bp PCR amplified product of *H. pylori ureC* gene generated 7 bands of different sizes that were used later for sorting 50 *H. pylori* strains into fifteen different *H. pylori* genotypes. The results of our study were similar to Menoni et al. who reported twelve digestion patterns by *MboI*^70^, and dissimilar to both Navabakbar and Salehi^71^ and Ozbey et al.^72^ who reported only five patterns. The higher number of fragments by PCR-RFLP technique in our study could be attributed to specimen collection from the outpatient clinic.

Comparing ERIC-PCR with other typing methods as PCR-RFLP, biotyping and antibiogram typing, the higher specificity of ERIC-PCR over the other three typing methods was evident (Discriminatory index (D) of ERIC-PCR, PCR-RFLP, antibiogram and biotyping were 0.94, 0.91, 0.88 and 0.61 respectively). Our protocol showed that ERIC-PCR analysis is a highly discriminating technique for *H. pylori* infectious strains. This finding agrees with Colding et al. who reported higher discriminatory power of genotypic methods than phenotypic methods^16^.

## Patients and methods

### Patients

This observational study was reviewed and approved by The Research and Ethical Committees of Al-Azhar Faculty of Medicine and Al-Azhar University Hospitals, Cairo, Egypt. Two hundred and thirty-two dyspeptic patients were enrolled in the study over two years, from December 2022 to February 2024. Patients were 127 males and 105 females, with age range from 18 to 74 years. The study population was outpatients admitted to the upper GIT endoscopy units at Al-Kahira Al Fatimya (n = 52), Al Matariya (n = 126) and Nasr City governmental hospitals (n = 54). Clinical biopsies were collected from patients by the attending physician. Informed consent was obtained from all participants and/or their legal guardian (s) before enrollment. As well, informed consent was obtained from the attending physician. Patients enrolled represented 7 governorates of Egypt. History of all patients was recorded and a full clinical examination with special emphasis on dyspepsia and the gastrointestinal system was carried out. The questionnaire form used in this study is described in Appendix C. Laboratory investigations including liver function tests (Alanine aminotransferase (ALT), Aspartate transaminase (AST), serum albumin, serum bilirubin), renal function tests (Blood Urea Nitrogen (BUN), serum creatinine), Prothrombin time (PT), Partial Thromboplastin Time (PTT), Complete Blood Count (CBC) if indicated and endoscopy results were carried out for each patient.

### Inclusion criteria

Patients suffering from epigastric pain, abdominal pain, and discomfort change in bowel habits, weight-loss, loss of appetite, nausea, and/or vomiting, melena and bloating.

### Exclusion criteria

Patients who rejected the enrollment; or who had history of sclerotherapy or band ligation of esophageal varices; patients with chronic liver disease and/or hepatocellular carcinoma (HCC); patients with portal splenic or hepatic vein thrombosis; patients with severe cardiac, chest or renal disease; patients prescribed H2 antagonist, PPI, antibiotics, non-steroidal anti-inflammatory drugs (NSAIDs) four weeks prior to endoscopy.

## Methods

### Collection of gastric biopsies

Three biopsies from the antrum and the corpus were taken from patients who undertook upper GIT endoscopy according to Nederskov-Sorensen et al., to overcome patchy distribution of *H. pylori* within gastric mucosa^73^. Specimens were collected using Olympus Videotrolley tv-z CLE-10 endoscopy machine, USA, and were kept immediately at 4 °C. Biopsies are collected with 1 ml of Brain Heart Infusion (BHI) broth (Oxoid, UK) in 5 ml screw cap test tube^74^ aseptically, and were immediately transported in ice box at 4 °C to the University’s local microbiology laboratory for immediate analyses. Tryptic Soy Broth (TSB) at 4 °C was used as a transport medium^8^.

### Selection of *H*. *pylori* isolates

A total of 50 *H. pylori* isolates presenting the studied clinical gastro-duodenal disorders as follows: GERD (5), gastric ulcer (GU, 10), Duodenal ulcer (DU, 5), Duodenitis (5), Gastritis (20), Normal mucosa (5) were subjected to phenotypic and genotypic characterization.

### Isolation of *H*. *pylori* strains

*H. pylori* strains were isolated and identified as has been described in Owen and Desai^75^. Under aseptic conditions, specimens temperatures were measured and time spent was recorded. Individual specimens were homogenized using sterile glass rod with vigorous shaking to release the organism from mucosal cells. Isolates were grown on selective media, i.e. BBA, BHI agar and CBA supplemented with 7% sheep blood and *H. pylori* selective supplement (Dent), Oxoid, UK^12^, and non-selective media, i.e. CCA supplemented with 7% sheep blood. The inoculated plates are incubated at 37 °C for 57 days under microaerophilic condition (5% O_2_, 10% CO_2_ and 85% N_2_) with 95% humidity using moistened cotton piece in anaerobic Jar with GasPak EZ Campy (Becton Dickinson, USA)^8^. A detailed description of different culture media used for *H. pylori* isolation is shown in Appendix C.

### Identification of *H. pylori* strains

Strains’ identification was based on (i) macroscopic examination, i.e. morphology of colonies including shape, texture, margin, and size. (ii) Microscopic examination of fresh antral biopsies using simple stain, i.e.: Leoffler's methylene blue stain after Misra et al.^76^. Briefly, a biopsy was removed from urea solution and imprint smears were made by slightly rolling it on a clean glass slide, using a hypodermic needle, the imprint smear was air dried and fixed in absolute methanol and stained by Leoffler's methylene blue stain. Slides were read against the oil immersion objective for *bacilli* with S and U shapes. As well, strains were isolated from fresh culture media using Gram stain after Harely and Prescott^77^. Briefly, a bacterial smear was prepared by methanol, crystal violet was applied**,** the iodine solution is added to form crystal violet-iodine complex. Ethanol is added for decolonization, and finally safranin. Slides were read against the oil immersion objective for red curved and straight rods. (iii) Biochemical identification including: catalase, oxidase, urease, *Campylobacter*-like organism tests (CLO), hydrogen bisulfide (H2S) production, nitrate reduction, indole, hippurate hydrolysis, ultra-rapid urease tests (URUT), under different conditions of growth; i.e. with 3.5% NaCl, with 1% glycine, at varying temperatures (25 °C and 42 °C), and susceptibility to cephalothin and nalidixic acid were performed after Al-Sulami et al.^27^. A detailed description of the biochemical procedures used for identification of *H. pylori* is shown in (Appendix D).

### Antimicrobial susceptibility testing

Antimicrobial susceptibility testing was performed using two methods: (1) MICs by agar dilution according to Mégraud and Lehours^38^; and (2) standard disc diffusion method using 10 antimicrobial agents currently in use in eradication therapy^6,39^.

### Antimicrobial susceptibility testing using MICs method

Antimicrobial susceptibility testing was performed by agar dilution method. Mueller–Hinton agar supplemented with Sheep’s blood (5% v/v) is used to prepare plates with different antibiotic concentrations. Range of antibiotics concentrations used is shown in (Supplementary Table S.T.6). Plates were inoculated with an inoculum corresponding to the McFarland 4 equivalent opacity standard, prepared from a 2-day old culture that was grown on a blood agar plate as described by Ogata et al.^59^. See Appendix C for the recipe of Turbidity Standard, McFarland 3.0^78^. Plates were then incubated at 37 °C for 72 h under sufficient microaerophilic conditions, and were read thereafter^24^. Interpretation of the susceptibility was done according to The European Committee on Antimicrobial Susceptibility Testing Breakpoint tables for interpretation of MICs and zone diameters EUCAST. Version 14.0, 2024^79^, as shown in (Supplementary Table S.T.7). Powders used and their sources are listed in Supplementary Data S.D. Table 1.

### Antimicrobial susceptibility testing by standard disc diffusion method

All isolates were tested against ten different groups of antimicrobial drugs from (Oxoid, UK), according to the method of Bauer and Kirby^80^. Discs of antimicrobial agents were stored at 4 °C and were brought to room temperature before usage. Discs and their potencies are listed in Supplementary Data S.D. Table 2. A suspension equal to the McFerland tube No. 3 was prepared for each isolate. Antimicrobial discs were aseptically placed onto the dried surface of inoculated Muller Hinton’s 15 cm agar plates supplemented with (7.0%) Sheeps’ blood. Plates were incubated at 37 °C under microaerobic conditions. Zones of inhibition were read after 48 h of incubation in mm using a ruler. The National Committee for Clinical Laboratory Standards (CLSI)^81^ did not approve the breakpoints: resistant; susceptible or intermediate resistant for interpreting data, therefore, we used breakpoints as previously published studies with a similar methodology^6,39^. Zones sizes and the reference for each antimicrobial agent’s breakpoint are listed in Supplementary Data S.D. Table 3.

### Phenotypic characterization of *H. pylori* strains

#### Biotyping

Fifty *H. pylori* strains were characterized on the basis of performed enzyme production using Analytical Profile Index (API ZYM system, BioMèrieux, France)^64^. The API ZYM strip is composed of 20 cupules, designed for the study of enzymatic reactions. The base of the strip, containing synthetic substrates allows enzymatic reactions to take place through non-woven fibers. The enzymatic tests are inoculated with a dense suspension of organisms, which is used to rehydrate the enzymatic substrates. The metabolic end-products produced during the incubation period are detected through colored reactions revealed by the addition of reagents.

### Preparation of the strip

Five ml of distilled water or demineralized water [or any water without additives or chemicals which may release gases (e.g. Cl_2_, CO_2_, etc.)] is added to an incubation box (tray and lid) into the honey-combed wells of the tray to create a humid atmosphere. Reference on the elongated flap of the tray is marked as it is stable. The strip is taken off and placed into the incubation box.

### Inoculation of the strip

Using a pipette or PSIpette, 65 μl of specimen is dispensed into each cupule. After inoculation, the plastic lid is placed on the tray and incubated for 4–4 ½ h at 37 °C, avoiding subjecting the inoculated strip to bright light.

### Reading the strip

One drop of ZYM A reagent (2.5 g of Tris, 1.1 ml of 37% HCI, 1 g of lauryl sulphate and 10 ml of distilled water) and 1 drop of ZYM B reagent are added to each cupule. By placing a surface-active agent (ZYM A reagent) in the cupule, the ZYM B reagent (35 mg of fast blue BB and 10 ml of methoxyethanol) dissolves. The color develops for at least 5 min. A value ranging from 0–5 is assigned corresponding to the color developed: 0 corresponds to a negative reaction, 5 to a reaction of maximum intensity and values 1, 2, 3 or 4 are intermediate reactions depending on the level of intensity (3, 4 or 5 as positive reactions). The resulting color intensities developed in each cupule were read against the API ZYM color chart. (Biomerieux, France) (Supplementary Table S.T.3). The performed enzyme profiles of *H. pylori* strains is identified by scheme published in Matsumoto et al.^25^.

### Molecular characterization of *H. pylori*

#### DNA extraction

Genomic DNA from *H. pylori* isolates was prepared from freshly harvested bacterial cells that were grown on 7% sheep’ blood agar, and incubated for 3 days at 37 °C under the optimal microaerophilic conditions. DNA was then extracted using ZR fungal/bacterial DNA MiniPrep kit from (ZYMO Research, Canada) according to the manufacturer’s instructions. Ultra-pure DNA yield of a 100 mg bacterial cells is obtained as described in Supplementary data SD Section 9.

#### Oligonucleotides

ERIC primers (ERIC-1R and ERIC-R) were used for detection of interspersed ERIC sequences within *H. pylori* genome as described in Hussain et al.^68^. *UreC* (GlmM) gene in *H. pylori* encodes a phosphoglucosamine mutase. The *UreC* gene was amplified using *ureC*-U and *ureC*-L primers described in Navabakbar and Salehi^71^. Sequences of the primers are shown in (Supplementary Table S.T.8A).

### Enterobacterial repetitive intergenic consensus (ERIC–PCR)

ERIC-PCR fingerprinting was carried out according to the method described by Sigh et al.^82^. The PCR reactions containing 5.0 μl of DNA, 25.0 μl Dream Taq green PCR master mix (2×), 30 pM each forward and reverse primers (Supplementary Table S.T.9A), and 2 U of Taq DNA polymerase (Thermo Scientific, Canada). The total volume was adjusted to 50 μl using autoclaved Milli-Q water. The reaction was performed using thermocycler over 35 cycles. Initial denaturation at 94 °C for 5 min; denaturation at 90 °C for 30 s.; annealing at 49 °C for 1 min; extension at 70 °C for 5 min; final extension at 70 °C for 10 min. The amplified products were then separated on a 1% agarose gel in 1× TAE buffer (40 mM Tris–acetate, 1 mM EDTA, pH 8.0) containing ethidium bromide (0.5 μg/ml), visualized under UV light.

### PCR-RFLP

The PCR-RFLP technique for genotyping of *H. pylori* strains was performed according to Navabakbar and Salehi^71^. An amplification reaction contained 10 pM of each primer (Supplementary Table S.T.8B), 25.0 μl of 2× PCR master mix solution (i-TaqTM), 5.0 μl of template DNA and the final volume was adjusted to 50 μl using bidistilled water (Supplementary Table S.T.9B). The reaction was performed using thermocycler (Biometra, USA), over 35 cycles. Initial denaturation at 94 °C for 5 min; denaturation at 90 °C for 30 s; annealing at 58 °C for 1 min; extension at 70 °C for 5 min; final extension at 70 °C for 10 min. Subsequently the amplified products were loaded on 1.5% agarose gel containing 0.5 μg ethidium bromide/ml gel, electrophoresed for 30 min at 100 V, and then visualized under UV light.

### Restriction digestion of amplified *ureC* gene

Fifteen μl of purified PCR products were digested with 10 U of MboI restriction enzyme separately in solution recommended by the manufacturer for 2 h at 37 °C. Ten μl of the digested products was analyzed by electrophoresis on 2.5% agarose gels (Gibco), and the corresponding bands were examined under UV light and compared with a standard 100 bp ladder for size estimation. The amplified products were separated on a 1% agarose gel in 1× TAE buffer (40 mM Tris–acetate, 1 mM EDTA, pH 8.0) containing ethidium bromide containing 0.5 μg/ml gel. The DNA was then visualized under UV light, Figs. 2 and 3.

### Statistical analysis

The collected data were coded, tabulated, and statistically analyzed using Statistical Package for Social Sciences software (SPSS) version 18.0, IBM Corp., Chicago, USA, 2009. Cross tabulation of variables was generated. The Chi square was used to detect statistically significant correlation among variables. The level of significance at *p* value < 0.05 is significant, otherwise is non-significant.

Simpsons index of diversity (The discriminatory index (D)) is based on the probability that two unrelated isolate samples of the test population will be placed into different typing groups. Simpsons index of diversity ranges from 0.0 to 1.0, where 1.0 indicates that a typing method can distinguish each member of a population from all other members of that population and, conversely, 0.0 indicates that all members of a strain population are of an identical type. The discriminatory index (D) is calculated using the formula described in Appendix B^83.^

### Limitations and future directions

In Egypt, high prevalence of multidrug resistant *H. pylori* underscores the urgent need for infection control measures to limit the spread of such bacteria. Studies should be conducted for subtyping of *H. pylori* using reliable and more powerful molecular methods such as PFGE, AFLP and plasmid profile analysis. Some clinical digestive disorders including gastritis, gastric ulcer, duodenal ulcer, duodenitis and GERD can correlate with different strains of *H. pylori* using different phenotypic and genotypic methods. Further studies are required to detect the possible sources of *H. pylori* infection especially drinking water. Such studies should investigate factors affecting the ability of *H. pylori* to survive in distribution systems and be isolated from drinking water, such as the bacterial strains, density of bacteria in the distribution systems, type of water pipe materials, efficiency of disinfection process and the techniques and materials used for culture. Furthermore, longitudinal studies are required to explain the relationship between *H. pylori* strains and resistance to antibiotics and to update the regimens of therapy. Population studies with bigger sample size and post-discharge follow up to early prognose gastro-duodenal cancers are required. Therapeutic modalities such as probiotics adjunct to prophylaxis of *H. pylori* infection, medicinal plants, lactoferrin in combination with antibiotic therapy or passive immunization with orally administered immunoglobulins, i.e. bovine colostrum have been tried as alternatives along with antibiotics, and worth longitudinal surveillance to prove efficacy.

## Conclusion and significance of the study

The present study elucidated the primary state of *H. pylori* isolates from Egyptian patients regarding their susceptibility to currently used antimicrobial drugs. It brings attention to establish a designed plan for antimicrobials prior to launching of treatment, in order to accomplish better integration between microbiological assessment and clinical usage of antimicrobials on a broad scale and regular basis. This could contribute to further successful eradication of *H. pylori*, thus minimizing risk of chronicity and consequently malignant transformation and sequential complications. The eradication therapy of *H. pylori* can contribute to a decrease in *H. pylori*-related diseases. Phenotypic characterization methods including biotyping and antibiogram typing had low discriminatory power, and accordingly genetic variations among *H. pylori* isolates were not evident. The genotyping techniques described in this study including ERIC-PCR and PCR-based RFLP presented rapid, sensitive and relatively simple tools, and acquired high discriminatory power for identifying infectious *H. pylori* sub-types. Such techniques are useful molecular tools for a rapid identification in a routine clinical microbiology laboratory. The present work highlights the need of a constant surveillance of *H. pylori* antibiotic resistance, so that tailoring therapy is feasible for clinical practice.

## Supplementary Information

Supplementary Information 1.

Supplementary Information 2.

Supplementary Information 3.

Supplementary Information 4.

Supplementary Information 5.

Supplementary Information 6.

## Data Availability

All data generated during this study are included in the published article. Source files can be provided after permission of the corresponding author.
