# Supplementary material for: PCR-based RFLP and ERIC-PCR patterns of Helicobacter pylori strains linked to multidrug resistance in Egypt
Source: Sci Rep. 2024 Sep 27;14:22273. doi: 10.1038/s41598-024-72289-z (PMC11436738; doi:10.1038/s41598-024-72289-z)
Supplement: Supplementary file 1 — Supplementary Information 1. [file 41598_2024_72289_MOESM1_ESM.docx]

**APPENDIX A**

**1. Antimicrobial susceptibility patterns**

**1.1. Frequency of antimicrobial susceptibility among *H. pylori* isolates by serial two-fold agar dilution method.**

| **Isolate**  **No.** | **MTZ** | **AM** | **CLA** | **E** | **TET** | **RD** | **LEV** | **CIP** | **FX** | **GM** |
| --- | --- | --- | --- | --- | --- | --- | --- | --- | --- | --- |
|  | R | R | R | S | R | S | S | S | S | S |
|  | R | R | R | S | R | S | S | S | S | S |
|  | R | R | R | S | R | S | S | S | S | S |
|  | S | S | R | S | S | S | R | S | S | R |
|  | R | S | R | S | S | S | S | S | S | S |
|  | R | R | S | R | S | R | S | S | R | R |
|  | R | S | R | S | S | S | S | S | S | S |
|  | R | R | I | R | R | S | S | R | S | R |
|  | R | R | S | R | R | R | S | R | R | R |
|  | R | R | I | R | R | R | S | S | R | R |
|  | S | S | R | S | S | S | S | S | S | S |
|  | S | R | R | R | S | R | S | R | S | S |
|  | R | R | S | R | R | R | S | R | R | R |
|  | R | S | R | S | S | S | S | S | S | S |
|  | S | R | S | S | S | S | S | S | S | S |
|  | R | S | R | S | R | S | S | S | S | S |
|  | R | S | R | S | S | S | S | S | S | S |
|  | S | R | S | S | S | S | S | S | S | S |
|  | R | R | R | R | R | R | R | R | R | R |
|  | S | R | R | R | S | R | S | R | S | S |
|  | R | S | R | S | S | S | S | S | S | S |
|  | R | R | S | S | S | S | S | S | S | S |
|  | R | S | R | S | R | S | S | S | S | S |
|  | R | S | S | S | S | S | R | S | R | S |
|  | R | R | R | R | R | R | S | R | S | S |
|  | R | R | R | S | R | S | S | S | S | S |
|  | R | R | I | S | R | S | R | S | R | R |
|  | R | R | S | S | S | S | S | S | S | S |
|  | R | R | S | S | S | S | S | R | R | S |
|  | R | R | S | R | S | R | S | S | S | R |
|  | R | R | I | S | S | R | S | S | S | S |
|  | R | R | S | S | S | S | S | S | S | S |
|  | R | S | S | S | S | S | R | S | R | S |
|  | R | R | I | S | R | R | S | S | S | S |
|  | S | R | R | R | S | R | S | R | S | S |
|  | R | R | S | S | R | S | S | R | R | S |
|  | S | S | R | S | S | S | R | S | S | R |
|  | R | S | R | S | R | S | S | S | S | S |
|  | R | R | S | S | S | S | S | S | S | S |
|  | R | R | S | S | S | S | S | S | R | R |
|  | R | R | S | S | S | S | S | S | S | S |
|  | R | S | R | S | S | R | R | S | S | S |
|  | R | R | R | S | R | S | S | S | S | S |
|  | S | S | R | S | S | S | S | S | S | S |
|  | S | R | S | S | S | S | S | S | S | S |
|  | R | S | S | S | S | S | R | S | S | R |
|  | S | R | R | R | S | R | S | S | S | S |
|  | R | R | R | S | R | S | S | S | S | S |
|  | R | S | S | S | S | S | R | S | R | S |
|  | S | R | S | S | S | S | S | S | S | S |
|  | S | R | S | S | S | S | S | S | S | S |
|  | S | S | R | S | S | S | R | S | S | R |
|  | R | R | R | R | R | R | R | R | R | R |
|  | R | R | S | S | S | S | S | S | S | S |
|  | R | R | S | S | S | S | S | S | S | S |
|  | S | R | S | S | S | S | S | S | S | S |
|  | R | R | S | S | S | S | S | R | S | R |
|  | R | R | S | S | R | R | R | R | S | S |
|  | R | S | R | S | S | S | S | S | S | S |
|  | R | R | S | S | R | S | S | S | S | R |
|  | S | S | R | S | S | S | S | S | S | S |
|  | R | R | I | S | R | S | S | S | S | R |
|  | R | S | R | S | S | S | S | S | S | S |
|  | R | R | S | S | R | R | S | S | S | S |
|  | R | R | S | S | S | S | S | S | S | S |
|  | R | S | R | S | S | S | S | S | S | S |
|  | R | R | R | R | R | R | R | R | R | R |
|  | R | S | S | S | S | S | R | S | R | S |
|  | R | S | R | S | S | S | S | S | S | S |
|  | S | S | R | S | S | S | R | S | S | R |
|  | R | R | S | S | R | R | S | R | R | R |
|  | R | R | R | S | S | S | S | S | S | S |
|  | R | R | S | S | S | S | S | S | S | S |
|  | R | R | R | S | R | S | S | S | S | S |
|  | R | S | S | S | S | S | R | S | R | S |
|  | S | S | R | S | S | S | R | S | S | R |
|  | R | R | S | S | S | S | S | S | S | S |
|  | R | R | S | S | S | S | S | S | S | S |
|  | S | S | R | S | S | S | R | S | S | R |
|  | R | S | S | S | S | S | R | S | S | R |
|  | R | S | S | S | S | S | R | S | R | S |
|  | R | R | S | S | S | S | S | S | S | S |
|  | R | R | S | S | S | S | S | S | S | S |
|  | R | S | R | S | S | S | S | S | S | S |
|  | R | R | S | S | S | S | S | S | S | S |
|  | S | S | R | S | S | S | S | S | S | S |
|  | S | R | S | S | S | S | S | S | S | R |
|  | R | S | S | S | S | S | R | S | R | S |
|  | R | R | I | R | S | R | R | S | R | R |
|  | R | S | R | S | R | S | S | R | S | S |
|  | R | R | S | S | R | R | S | S | R | R |
|  | R | R | I | S | R | S | S | S | R | S |
|  | R | R | S | S | S | S | S | S | S | S |
|  | R | R | S | R | R | S | S | S | S | R |
|  | R | S | S | S | S | S | R | S | S | R |
|  | R | R | S | S | R | S | S | S | R | R |
|  | R | R | S | S | S | S | S | S | S | S |
|  | R | R | R | S | S | S | S | S | S | S |
|  | R | R | S | R | S | R | S | R | R | R |
|  | S | S | R | S | S | S | S | S | S | S |
|  | R | R | S | S | S | S | S | S | S | S |
|  | R | R | R | R | R | R | R | R | R | R |
|  | R | R | R | S | S | S | S | S | S | S |
|  | R | R | S | S | R | R | S | S | R | R |
|  | R | R | R | S | S | S | S | S | S | S |
|  | S | R | R | R | R | S | S | S | S | S |
|  | R | R | S | S | S | S | S | S | S | S |
|  | R | R | S | S | S | S | S | S | S | S |
|  | R | S | S | S | R | S | S | R | S | S |
|  | S | R | S | S | S | S | S | S | S | S |
|  | R | R | R | S | S | S | S | S | S | S |
|  | R | R | S | S | S | S | S | S | S | S |
|  | R | R | I | S | R | S | R | R | S | S |
|  | R | S | S | S | S | S | R | S | S | R |
|  | R | R | S | S | S | S | S | S | S | S |
|  | R | R | S | R | R | S | S | S | R | R |
|  | R | R | S | S | S | S | S | S | S | S |
|  | R | R | I | S | R | R | S | R | R | S |
|  | R | S | S | S | S | S | R | S | S | R |
|  | R | S | R | S | S | S | S | S | S | S |
|  | R | R | S | R | R | R | S | S | S | R |
|  | R | R | S | S | S | S | S | S | R | S |
|  | R | R | S | S | S | S | S | S | R | R |
|  | R | R | R | S | S | S | S | S | S | S |
|  | R | R | R | S | R | S | S | S | S | S |
|  | R | R | R | S | R | S | S | R | S | S |
|  | R | R | S | S | S | S | S | S | S | S |
|  | R | S | R | S | S | S | S | S | S | S |
|  | S | S | R | S | S | S | S | S | S | S |
|  | R | R | I | S | S | S | S | S | S | R |
|  | R | S | S | S | R | S | S | R | S | S |
|  | R | R | S | S | S | S | S | S | S | S |
|  | R | R | S | S | S | S | S | S | S | S |
|  | R | S | S | S | S | S | R | S | R | S |
|  | S | S | R | S | S | S | S | S | S | S |
|  | R | R | S | R | R | R | R | R | S | R |
|  | R | R | R | S | R | S | S | S | S | S |
|  | R | R | R | S | S | S | S | S | S | S |
|  | R | R | R | S | S | S | R | S | R | S |
|  | R | R | R | S | S | S | S | S | S | S |
|  | R | S | R | S | S | S | S | S | S | S |
|  | S | S | R | S | S | S | R | S | S | R |
|  | R | S | S | S | S | S | R | S | R | S |
|  | R | R | I | S | S | R | S | S | R | R |
|  | R | S | R | S | S | S | S | S | S | S |
|  | R | S | S | S | S | S | R | S | S | R |
|  | S | S | R | S | S | S | S | S | S | S |
|  | R | R | S | S | S | S | S | S | S | S |
|  | R | R | S | R | S | S | S | R | R | R |
|  | R | S | R | S | S | S | S | S | S | S |
|  | R | R | I | S | R | S | S | R | S | R |
|  | R | R | S | S | R | S | S | S | R | R |
|  | R | R | S | S | R | S | S | S | S | S |
|  | R | S | R | S | S | S | S | S | S | S |
|  | R | R | R | S | S | S | S | S | S | S |
|  | R | R | S | R | S | R | S | S | R | R |
|  | R | R | R | S | S | S | S | S | S | S |
|  | R | R | S | S | S | R | S | R | R | R |
|  | R | S | R | S | R | S | S | R | S | S |
|  | R | R | S | S | S | S | S | S | S | S |
|  | R | R | S | S | S | S | S | S | S | S |
|  | R | S | R | S | S | S | R | S | R | S |
|  | R | R | S | S | S | S | S | S | S | S |
|  | R | S | S | S | R | S | S | R | S | S |
|  | R | S | S | S | R | S | S | R | S | S |
|  | R | S | S | S | R | S | S | R | S | S |
|  | R | S | R | S | S | S | S | S | S | S |

**1.2. Frequency of antimicrobial susceptibility among *H. pylori* isolates by standard disc diffusion method.**

| **Isolate**  **No.** | **MTZ** | **AM** | **CLA** | **E** | **TET** | **RD** | **LEV** | **CIP** | **FX** | **GM** |
| --- | --- | --- | --- | --- | --- | --- | --- | --- | --- | --- |
|  | R | R | R | S | R | S | S | S | S | S |
|  | R | R | S | S | R | R | S | S | S | S |
|  | R | R | S | S | S | S | S | S | S | S |
|  | R | R | S | S | S | S | S | S | S | S |
|  | S | R | R | R | S | R | S | R | S | S |
|  | R | S | S | R | S | R | S | S | R | R |
|  | R | S | R | S | S | S | S | S | S | S |
|  | R | R | I | R | R | S | S | S | S | R |
|  | R | R | S | R | R | R | S | R | R | R |
|  | R | R | I | R | R | R | S | S | R | R |
|  | S | S | R | S | S | S | S | S | S | S |
|  | R | S | R | S | S | S | S | S | S | S |
|  | R | S | S | R | R | R | S | R | R | R |
|  | R | S | R | S | S | S | S | S | S | S |
|  | S | R | S | S | R | S | S | S | S | S |
|  | R | S | R | S | R | R | S | S | S | S |
|  | R | S | R | S | S | S | S | S | S | S |
|  | S | R | S | S | S | S | S | S | S | S |
|  | R | R | R | R | R | R | R | R | R | R |
|  | S | R | S | R | R | R | S | S | S | S |
|  | S | S | R | S | S | S | S | S | S | S |
|  | R | S | S | S | S | S | R | S | R | S |
|  | R | S | S | S | R | S | S | S | S | S |
|  | R | S | S | S | R | R | S | S | S | R |
|  | R | R | R | R | R | R | S | S | S | S |
|  | R | S | R | S | R | S | S | S | S | S |
|  | R | R | I | S | R | S | R | S | R | R |
|  | S | S | R | S | S | S | R | S | S | R |
|  | R | R | S | S | R | R | S | R | R | S |
|  | R | R | S | R | S | R | S | S | S | R |
|  | R | S | I | S | R | R | S | S | S | S |
|  | R | S | S | S | S | S | R | S | R | S |
|  | R | S | S | S | S | S | R | S | R | S |
|  | R | R | I | S | R | R | S | S | S | S |
|  | R | R | R | S | R | S | S | S | S | S |
|  | R | R | S | S | R | S | S | R | R | S |
|  | R | R | R | R | R | R | R | R | R | R |
|  | R | S | R | S | R | S | S | S | S | S |
|  | R | R | S | S | S | S | S | S | S | S |
|  | R | R | S | S | R | S | S | S | R | R |
|  | R | S | R | S | S | S | S | S | S | S |
|  | R | S | R | S | S | R | S | S | S | S |
|  | R | R | S | S | R | R | S | S | S | S |
|  | R | R | R | R | R | R | R | R | R | R |
|  | S | R | S | S | S | S | S | S | S | S |
|  | R | S | S | S | S | R | S | S | S | R |
|  | R | S | R | S | S | S | S | S | S | S |
|  | R | S | S | S | S | S | R | S | R | S |
|  | S | R | S | S | S | S | S | S | S | S |
|  | S | R | S | S | S | S | S | S | S | S |
|  | S | S | R | S | S | S | S | S | S | S |
|  | S | R | R | S | S | S | S | S | S | S |
|  | R | R | R | S | R | S | S | S | S | S |
|  | R | R | S | S | S | S | S | S | S | S |
|  | R | R | S | S | S | S | S | S | S | S |
|  | R | S | R | S | S | S | S | S | S | S |
|  | R | R | S | S | S | R | S | R | S | R |
|  | R | R | S | S | R | R | S | S | S | S |
|  | S | R | R | R | S | R | S | R | S | S |
|  | S | R | S | S | R | R | S | S | S | R |
|  | R | S | R | S | S | S | S | S | S | S |
|  | R | R | I | S | R | S | S | S | S | R |
|  | R | R | S | S | S | S | S | S | S | S |
|  | R | R | S | S | R | R | S | S | S | S |
|  | R | R | S | S | S | S | S | S | S | S |
|  | R | R | R | S | R | S | S | S | S | S |
|  | R | S | S | S | S | S | R | S | R | S |
|  | S | R | R | R | S | R | S | R | S | S |
|  | R | R | S | S | S | S | S | S | S | S |
|  | S | S | R | S | S | S | S | S | S | S |
|  | R | R | S | S | R | R | S | R | R | R |
|  | R | R | R | S | S | S | S | S | S | S |
|  | R | S | S | S | S | R | S | S | S | S |
|  | R | S | S | S | S | S | R | S | R | S |
|  | R | S | S | S | S | S | R | S | R | S |
|  | S | S | S | S | S | S | R | S | S | R |
|  | R | R | S | S | S | S | S | S | S | S |
|  | S | S | R | S | S | S | R | S | S | R |
|  | S | R | S | S | S | S | S | S | S | S |
|  | R | S | S | S | S | S | R | S | S | R |
|  | R | R | S | S | S | S | S | S | S | S |
|  | R | S | S | S | S | S | R | S | R | S |
|  | S | S | R | S | S | S | S | S | S | S |
|  | S | S | R | S | S | S | R | S | S | R |
|  | R | R | S | S | S | S | S | S | S | S |
|  | R | S | R | S | S | S | S | S | S | S |
|  | S | R | S | S | S | S | S | S | S | R |
|  | R | R | S | S | S | S | S | S | S | S |
|  | R | R | I | R | S | R | R | S | R | R |
|  | R | S | S | S | R | S | S | R | S | S |
|  | R | R | S | S | R | R | S | S | S | R |
|  | R | R | I | S | R | S | S | S | R | S |
|  | R | R | S | S | S | S | S | S | S | S |
|  | R | R | S | R | R | S | S | S | S | R |
|  | R | S | S | S | S | S | R | S | S | R |
|  | R | R | S | S | R | S | S | S | S | S |
|  | R | R | S | S | S | S | S | S | S | S |
|  | R | R | S | S | S | S | S | S | S | S |
|  | R | R | S | R | S | R | S | R | R | S |
|  | R | R | S | S | S | S | S | S | S | S |
|  | R | R | S | S | S | S | S | S | S | S |
|  | R | R | S | R | R | R | R | R | R | R |
|  | R | R | S | S | S | S | S | S | S | S |
|  | R | R | S | S | R | R | S | S | S | R |
|  | R | R | S | S | S | S | S | S | S | S |
|  | S | R | R | R | R | S | S | S | S | S |
|  | R | R | S | S | S | S | S | S | S | S |
|  | R | S | S | S | S | S | S | S | S | S |
|  | R | S | S | S | R | S | S | R | S | S |
|  | S | R | S | S | S | S | S | S | S | S |
|  | R | R | S | S | S | S | S | S | S | S |
|  | R | R | S | S | S | S | S | S | S | S |
|  | R | R | I | S | R | S | R | R | S | S |
|  | R | S | S | S | S | S | R | S | S | R |
|  | R | R | S | S | S | S | S | S | S | S |
|  | R | R | S | R | R | S | S | S | R | S |
|  | S | S | R | S | S | S | S | S | S | S |
|  | R | R | I | S | R | R | S | R | R | S |
|  | R | S | S | S | S | S | R | S | S | R |
|  | R | R | S | S | S | S | S | S | S | S |
|  | R | R | S | R | R | R | S | S | S | S |
|  | R | R | S | S | S | S | S | S | S | S |
|  | R | R | S | S | S | S | S | S | R | S |
|  | R | R | R | S | S | S | S | S | S | S |
|  | R | S | R | S | S | S | S | S | S | S |
|  | R | R | R | S | R | S | S | R | S | S |
|  | R | R | S | S | S | S | S | S | S | S |
|  | R | S | R | S | S | S | S | S | S | S |
|  | S | S | S | S | S | S | S | S | S | S |
|  | R | R | I | S | S | S | S | S | S | R |
|  | R | S | S | S | R | S | S | R | S | S |
|  | R | R | S | S | S | S | S | S | S | S |
|  | S | S | R | S | S | S | R | S | S | R |
|  | S | S | R | S | S | S | R | S | S | R |
|  | R | S | R | S | S | S | S | S | S | S |
|  | R | R | S | R | R | R | R | S | S | R |
|  | R | R | R | S | R | S | S | S | S | S |
|  | R | R | S | S | S | S | S | S | S | S |
|  | R | R | R | S | S | S | R | S | R | S |
|  | R | R | R | S | S | S | S | S | S | S |
|  | R | S | R | S | S | S | S | S | S | S |
|  | R | R | S | S | S | S | S | S | S | S |
|  | S | S | R | S | S | S | S | S | S | S |
|  | R | R | I | S | S | R | S | S | R | R |
|  | R | S | R | S | S | S | S | S | S | S |
|  | R | S | S | S | S | S | R | S | S | R |
|  | R | R | S | S | R | S | S | R | S | S |
|  | R | R | S | S | S | S | S | S | S | S |
|  | R | R | S | S | S | S | S | S | R | R |
|  | R | R | R | S | R | S | S | S | S | S |
|  | R | R | I | S | R | S | S | S | S | R |
|  | R | R | S | S | R | S | S | S | S | R |
|  | R | R | S | S | R | S | S | S | S | S |
|  | R | R | S | S | S | S | S | S | S | S |
|  | R | R | S | S | S | S | S | S | S | S |
|  | R | R | S | R | S | R | S | S | R | R |
|  | R | R | R | S | S | S | S | S | S | S |
|  | R | R | S | S | S | R | S | R | R | R |
|  | R | S | R | S | R | S | S | S | S | S |
|  | S | S | R | S | S | S | S | S | S | S |
|  | R | R | S | S | S | S | S | S | S | S |
|  | R | S | R | S | S | S | R | S | S | S |
|  | R | R | S | S | S | S | S | S | S | S |
|  | R | S | S | S | R | S | S | R | S | S |
|  | R | S | S | S | R | S | S | R | S | S |
|  | R | S | S | S | R | S | S | R | S | S |
|  | R | S | R | S | S | S | S | S | S | S |
