# Supplementary material for: PCR-based RFLP and ERIC-PCR patterns of Helicobacter pylori strains linked to multidrug resistance in Egypt
Source: Sci Rep. 2024 Sep 27;14:22273. doi: 10.1038/s41598-024-72289-z (PMC11436738; doi:10.1038/s41598-024-72289-z)
Supplement: Supplementary file 2 — Supplementary Information 2. [file 41598_2024_72289_MOESM2_ESM.docx]

**APPENDIX B**

**Calculation of discriminatory index (D):**

$$D=1-\frac{1}{N(N-1)} . \sum_{j=1}^{s} nj (nj-1)$$

where;

N = The total number of isolates in the sample population.

s = The total number of types described.

*nj* = The number of isolates belonging to the most numerous type.

For example in case of **ERIC-PCR** technique;

N = 50

s = 22

*nj* = 6

Accordingly;

$$D=1-\frac{1}{50(50-1)} . \sum_{j=1}^{22} 6 (6-1)$$

= 0.94
