# Supplementary material for: PCR-based RFLP and ERIC-PCR patterns of Helicobacter pylori strains linked to multidrug resistance in Egypt
Source: Sci Rep. 2024 Sep 27;14:22273. doi: 10.1038/s41598-024-72289-z (PMC11436738; doi:10.1038/s41598-024-72289-z)
Supplement: Supplementary file 3 — Supplementary Information 3. [file 41598_2024_72289_MOESM3_ESM.docx]

**APPENDIX C**

**Culture media**

1. **Ready-made media:**

Ready-made culture media used in this study are listed in Table (1). Media were prepared according to the manufacturer’s instructions and sterilized by autoclaving at 121°C for 20 minutes.

**Table 1. Ready-made culture media used in this study**

| Media | Sources |
| --- | --- |
| Columbia agar | HIMEDIA. Laboratories, PVT. Limited, India. |
| Brain heart infusion agara | Oxoid, UK. |
| SIM | Difco Laboratories. |
| Urea agar base | Difco Laboratories. |
| TSI | Oxoid, UK. |
| Brucella agar | HIMEDIA. Laboratories, PVT. Limited, India. |
| Tryptic soy broth | Oxoid, UK. |
| Brain heart infusion broth | HIMEDIA. Laboratories, PVT.Limited, India. |
| Mueller-Hinton agar | Oxoid, UK. |
| Blood agar base | HIMEDIA. Laboratories, PVT.Limited, India. |
| PSD | Difco Laboratories. |

1. **2. Laboratory prepared media:**

The laboratory prepared media included the following:

Selective brain heart infusion blood agar

Blood agar 5 %

Urease agar

Selective brucella blood agar

Selective Columbia blood agar

Selective brain heart infusion broth

Nitrate broth

Storage media

Mueller-Hinton agar blood agar

Selective tryptic soya broth

H. pylori selective supplement (Dent) was added to prepare culture media selective for growth of H. pylori. Composition and concentrations of (Dent) supplement are listed in Table (2).

**Table 2: H. pylori selective supplement (Dent)**

| Vial contents | per litre |
| --- | --- |
| Vancomycin | 10.0 mg |
| Trimethoprim | 5.0 mg |
| Cefsulodin | 5.0 mg |
| Amphotericin B | 5.0 mg |

**2.1. Selective brain heart infusion broth:**

Brain Heart Infusion powder……...................................................37.0 g/L

Dent supplement...........................................................................2 Vials/L

Sheep blood...................................................................................... 7.0 %

Distilled water up to..................................................................... 1000 ml

Final pH: 7.4 ± 0.2 at 25°C.

Dissolve 37 g of the medium in one liter of purified water then warm up with frequent agitation till complete dissolving, and finally autoclave at 121°C for 15 minutes. Cool to 50°C and add 7% sterile sheep blood. Add one vial of (Dent.) selective supplement to each 500 ml of prepared selective media.

**2.2. Selective tryptic soya broth:**

Tryptic soya broth powder….........................................................30.5 g/L

Dent supplement...........................................................................2 Vials/L

Sheep blood...................................................................................... 7.0 %

Distilled water up to..................................................................... 1000 ml

Final pH: 7.4 ± 0.2 at 25°C.

Dissolve 30 g of the medium in one liter of purified water then heat with frequent agitation to completely dissolve the medium, autoclave at 121°C for 15 minutes. Cool to 50°C and add 7% sterile sheep blood. Add one vial of (Dent.) selective supplement to each 500 ml of prepared selective media.

1. **3. Christensen urease agar:**

Christensen urea agar powder…………………….…………..…..24.0 g/L

Sterile urea (40%)………………………………..…………………..5.0 % Dis.

Water up to ………………………………………………...1000 ml

Final pH (at 25°C) 6.8 ± 0.2.

Suspend 24.01 g in 950 ml distilled water. Boil to dissolve the medium completely. Sterilize by autoclaving at 115°C for 20 minutes. Cool to 50°C and aseptically add 50 ml of sterile 40% Urea Solution and mix well. Dispense into sterile tubes and allow to set in the slanting position. Do not overheat or reheat the medium as urea decomposes very easily.

**2.4. Blood agar.**

Blood agar was made by adding 50 ml of sterile defibrinated sheep blood to 950 ml of blood agar base. Blood agar base was sterilized by autoclaving and allowed cool to 50ºC. Defibrinated blood was added aseptically. The agar was mixed and distributed into plates. Blood should never be added before autoclaving **(Cowan & Steel, 1993)**.

**2.5. Storage media** (**Oskouei *et al.,* 2010):**

Stock cultures were maintained in glycerol brain heart infusion broth (for long term preservation) in which 0.20 ml of glycerol (**Sigma, St. Louis, MO, U.S.A.)** sterilized by autoclaving for 20 min added to 0.80 ml of bacterial culture, vortex the culture to ensure that the glycerol was evenly dispersed.

**2.6. Nitrate broth (Colle & Marr, 1996)**:

KNO_3_ (nitrite free)............................................................................. 0.2 g

Peptone .............................................................................................. 5 g

Distilled water up to........................................................................... 1 L

These components are dispensed in 5ml in tubes and sterilized by autoclaving at 121ºC for 15 min.

**2.7. Selective brain heart infusion (BHI) blood agar:**

Brain Heart Infusion agar powder…..............................................47.0 g/L

Dent supplement............................................................................2 Vials/L

Sheep blood...................................................................................... 7.0 %

Distilled water up to..................................................................... 1000 ml

Final pH: 7.4 ± 0.2 at 25°C.

Dissolve 47 g of the medium in one liter of purified water, heat with frequent agitation to completely dissolve the medium and finally autoclave at 121°C for 15 minutes. Cool to 50°C and add 7% sterile sheep blood. Add one vial of (Dent.) selective supplement to each 500 ml of prepared selective media.

**2.8 Selective Columbia blood agar:**

Columbia blood agar base powder…….........................................39.0 g/L

Dent supplement............................................................................2 Vials/L

Sheep blood...................................................................................... 7.0 %

Distilled water up to..................................................................... 1000 ml

Final pH: 6.8 ± 0.2 at 25°C.

Dissolve 37 g of the medium in one liter of purified water then heat with frequent agitation to completely dissolve the medium and finally autoclave at 121°C for 15 minutes. Then Cool to 50°C and add 7% sterile sheep blood. Finally add one vial of (Dent.) selective supplement to each 500 ml of prepared selective media.

**2.9 Selective brucella blood agar:**

Brucella blood agar base powder…..............................................37.5 g/L

Dent supplement...........................................................................2 Vials/L

Sheep blood...................................................................................... 7.0 %

Distilled water up to..................................................................... 1000 ml

Final pH: 6.9 ± 0.2 at 25°C.

The culture medium was prepared by dissolving 37 g of the medium in one liter of purified water, heat up with frequent agitation to completely dissolve the medium, autoclave at 121°C for 15 minutes. Cool to 50°C and add 7% sterile sheep blood. Finally add one vial of (Dent.) selective supplement to each 500 ml of prepared selective media.

**2.10 Muller-Hinton blood agar**

powder…….................................................38.0 g/L

Dent supplement...........................................................................2 Vials/L

Sheep blood...................................................................................... 7.0 %

Distilled water up to..................................................................... 1000 ml

Final pH: 7.3 ± 0.2 at 25°C.

Dissolve 37 g of the medium in one liter of purified water, heat up with frequent agitation to completely dissolve the medium, autoclave at 121°C for 15 minutes. Cool to 50°C and add 7% sterile sheep blood. Add one vial of (Dent.) selective supplement to each 500 ml of prepared selective media.

**3. Reagents and stains.**

**3.1. Reagents for nitrate reduction test (Kauffmann, 1954)**.

**Solution A:**

8 g of sulfanilic acid was dissolved in 1L of 5N acetic acid (30 %) and stored in a glass stoppered brown bottle.

**Solution B:**

5 g of α-naphthylamine was dissolved in 1L of 5N acetic acid (30 %) and stored in a glass stoppered brown bottle.

Equal volumes of solutions A+B were mixed immediately before use to give the test reagent and 0.1 ml of the mixture is added to each tube. Positive reaction = red color within 10 minutes.

**3.2. Catalase reagent. (Macfaddin, 2000)**.

Add 1ml of 30 % H_2_O_2_ to 9 ml of distilled water, the final concentration of the solution was 3 %, then, it was stored in a dark sterile bottle.

**3.3. Oxidase reagent. (York *et al.,* 2004)**.

Add 0.1 g of N, N, N', N'-Tetramethyl-p-phenylenediamine**·**2HCl to 10 ml of Distilled water, finally, store in a dark sterile bottle.

**3.4. Phenol red reagent. (Yamaguchi *et al.,* 1997)**.

Dissolve 0.10 g of phenol red in 100 mL of alcohol, filter if necessary. Final pH 6.8–8.2

**3.5. Gram stain (Harley & Prescott, 2002).**

**Crystal violet:**

Crystal violet 85 % dye ...................................................................... 2.0 g

Ethyl alcohol (95 %) ......................................................................... 20 ml

**Gram’s iodine solution:**

Iodine crystals .................................................................................. 1.0 g

Potassium iodide .............................................................................. 2.0 g

Distilled H_2_O ................................................................................. 300 ml

**Safranin solution:**

Safranin ........................................................................................ 2.5 g

Ethyl alcohol (95%) ...................................................................... 100 ml

**3.6. Loeffler’s methylene blue stain (Murray *et al.* 1995)**

Methylene Blue, Certified………………………………..…………3.0 g

potassium Hydroxide, 10%...............................................................1.0 ml

Ethanol, 95%................................................................................300.0 ml

**3.7. Ninhydrin reagent (2%). (Carter *et al.,* 2008)**

Ninhydrin powder………………………………..…………………2.0 g

Distilled water …………………………………………………..100.0 ml

**3.8. Kovacs’ reagent for Indole (Hemraj *et al*., 2013)**.

Isoamyl alcohol ............................................................................... 150 ml

Conc. HCl .......................................................................................... 50 ml

P-dimethylaminobenzaldehyde (DMAB)......................................... 10 gm

DMAB was dissolved in the alcohol; gentle heating might be required to get the aldehyde into solution. The acid was then slowly added to the aldehyde-alcohol mixture. The mixture was stored in a brown glass bottle in the refrigerator at 4ºC and used immediately.

**3.9. 2% Agarose gel.**

Agarose……………………………………………………….……..1.0 g

Distilled water..……………………………………………………..50 ml.

**3.10. Ethidium bromide solution (0.05 mg/ml) (Ozdemir *et al*., 2013).**

0.5 mg of ethidium bromide **(Sigma)** was added to 10 ml of sterile distilled H_2_O, stirred on a magnetic stirrer for several hours to ensure that the dye has dissolved. The solution stored in dark bottle at room temperature.

**Caution**: Ethidium bromide is a powerful mutagen and moderately toxic. Gloves should be wear when working with solutions contain this dye.

1. **Chemicals**

4.1.1. Glycerol, ethanol 70 %, concentrated HCl, disodium hydrogen phosphate (Na2 HPO4), potassium acetate, potassium hydroxide (KOH), sodium chloride (NaCl), potassium nitrate (KNO3 nitrite free), potassium iodide, iodine crystals, methanol, barium chloride dihydrate (BaCl2.2H2O), 1 % sulfuric acid, hydrogen peroxide 30% and urea were supplied from El Nasr pharmaceutical chemicals Co., Cairo, Egypt.

Phenol red, isoamyl alcohol peptone, potassium chloride, tris base, tris-HCL, sodium acetate.3H2O, boric acid, bromophenol blue, sucrose, ethidium bromide and acetic acid were supplied from Sigma Aldrich Chemical Co. LTD, Germany.

Crystal violet, saffranin, and Leoffler & apos, methylene blue were supplied from Nice chemicals. Pvt. Ltd. India. Electrophoresis-grade agarose powder, tetramethylethylenediamine (TEMED) were supplied from GIBCO Bethesda Research Lab., U.S.A. yeast extract, and agar were obtained from Difco Laboratories.

**4.1.2. Tools:** All tools used in the current study i.e. Sterile cotton swabs, Ice box, test tubes, capped eppendorf tubes, screw capped wide-mouthed glass tubes, hypodermic needle, slides, Petri-dishes, membrane filter (0.22 μm), flasks, a rubber stopper Erlenmeyer sidearm flask, plastic syringes, anaerobic EZ GasPack campy, measuring cylinders, anaerobic jar, beakers and graduated pipettes were sterile, disposable.

**4.1.3. Instruments**

PCR Thermocycler (Biometra, USA)

Autoclave. (Hirayama Manufacturing Corporation, Japan)

Endoscopy machine (Olympus Videotrolley tv-z CLE-10 machine, USA)

Incubator (Heraeus, USA)

Vortex (Fisher Scientific)

Horizontal Gel Electrophoresis platform

Gel casting platform (BioRad)

Gel combs (Slot forms) (Bio-Rad)

DC power supply (GIBCO-BRL)

UV Ttransluminator (Fotodyne, Hartland, Wi, U.S.A.)

**4.2. Biological materials**

Sheep blood used in this study was obtained from Veterinary Serum and Vaccine Research Institute, Cairo. Egypt.

**4.3. Standard solutions**

**4.3.1. Turbidity standard (McFarland 3.0) (Baron & Fienegold, 1990):**

Turbidity standard (McFarland No. 0.5) was prepared by adding 3.0 ml of 1.175 % barium chloride dihydrate (BaCl2.2H2O) to 99.5 ml of 1 % sulfuric acid in graduated cylinder, 10 ml of mixture were put in sterile test tubes and stored in the dark at room temperature. The absorbance was measured by a spectrophotometer at a wavelength of 625 nm. The acceptable absorbance range for the standard is 0.08 - 0.13. The contents were mixed well prior to standardizing bacterial cells number which was equivalent to 1.5 × 10 8 CFU/ml.

**4.3.2. Normal saline,** **Hussein et al. (2013)**

Dissolve 4.24 g of NaCl in 500 ml of distilled water to obtain 0.145 M NaCl. Thereafter it was autoclaved and stored at 4^o^ C.

1. **Identifications kits**

5.1. **Oxidase discs**: Oxidase discs were obtained from HIMEDIA Laboratories, PVT. Limited, India.

5.2. **Hippurate discs** : Remel hippurate discs were obtained from Thermo Scientific™, USA.

**6. DNA work**

**6.1. Reagents for extraction of DNA of *H. pylori* isolates and their volumes**

| Reagent | Volume |
| --- | --- |
| Bacterial DNA Binding Buffer | 1200 μl |
| Lysis Solution | 750 μl |
| DNA Pre-Wash Buffer | 200 μl |
| Bacterial DNA Wash Buffer | 500 μl |
| DNA Elution Buffer | 100 μl |

1xTEA buffer (pH 8)

Electrophoresis buffer (Tris-borate-EDTA buffer) (TBE)

Phosphate buffer saline (PBS)

MboI Restriction enzyme

**6.2. Extraction of DNA from *H. pylori* isolates**

1. 100 mg (wet weight) bacterial cells that have been resuspended in up to 200 µl of isotonic phosphate buffer saline (PBS) to a ZR BashingBead™ Lysis Tube. Add 750 µl Lysis Solution added to the tube.
2. Secure in a bead beater fitted with a 2 ml tube holder assembly and process at maximum speed for 5 minutes.
3. Centrifuge the ZR BashingBead™ Lysis Tube in a microcentrifuge at 10,000 x g for 1 minute.
4. Transfer up to 400 µl supernatant to a Zymo-Spin™ IV Spin Filter (orange top) in a Collection Tube and centrifuge at 7,000 rpm for 1 minute. Snap off the base of the Zymo-Spin IV™ Spin Filter prior to use.
5. 1,200 µl of Bacterial DNA Binding Buffer added to the filtrate in the Collection Tube
6. Transfer 800 µl of the mixture from Step 5 to a Zymo-Spin™ IIC Column in a Collection Tube and centrifuge at 10,000 x g for 1 minute.
7. Discard the flow through from the Collection Tube and repeat the Step 4.
8. 200 µl DNA Pre-Wash Buffer added to the Zymo-Spin™ IIC Column in a new Collection Tube and centrifuge at 10,000 x g for 1 minute.
9. 500 µl Fungal/Bacterial DNA Wash Buffer added to the Zymo-Spin™ IIC Column and centrifuge at 10,000 x g for 1 minute.
10. Transfer the Zymo-Spin™ IIC Column to a clean 1.5 ml microcentrifuge tube and add 100 µl DNA Elution Buffer directly to the column matrix. Centrifuge at 10,000 x g for 30 seconds to elute the DNA. Ultra-pure DNA is now ready for subsequent PCR reactions.

**6.3. PCR oligonucleotides**

ERIC primers (ERIC-1R and ERIC-R) were used for detection of interspersed ERIC sequences within *H. pylori* genome (Hussien et al., 2004). UreC (GlmM) gene *in H. pylori*, which encodes a phosphoglucoseamine mutase, was amplified using ureC-U and ureC-L primers used for detection of ureC gene (Navabakbar and Salehi, 2004).

1. **Antibiogram typing**

**Antimicrobial agents used for antimicrobial susceptibility testing**

- 1. **Disc diffusion method**
     1. **Discs of antimicrobial agents and their potencies used for antimicrobial susceptibility testing of *H. pylori* isolates by Disc Difussion method.**

| Antimicrobial agent | Disc strength |
| --- | --- |
| Amoxicillin (AM) | 10 μg |
| Clarithromycin (CLA) | 15 μg |
| Metronidazole (MTZ) | 5 μg |
| Tetracycline (TE) | 30 μg |
| Levofloxacin (LEV) | 5 μg |
| Ciprofloxacin (CIP) | 10 μg |
| Erythromycin (E) | 15 μg |
| Furazolidone (FX) | 100 μg |
| Gentamicin (GM) | 10 μg |
| Rifampicin (RD) | 5 μg |

- - 1. **Zone sizes for each antimicrobial agent used for antimicrobial susceptibility testing of *H. pylori* isolates by Disc Difussion method.**

| Antimicrobial agent | Code | Zone size breakpoints (mm) | | | Reference |
| --- | --- | --- | --- | --- | --- |
|  |  | R | I | S |  |
| Amoxicillin | AM | >25 | - | ≥25 | (Ozbey *et al.,* 2012) |
| Clarithromycin | CLA | >30 | - | ≥30 | (Ozbey *et al.,* 2012) |
| Metronidazole | MTZ | >16 | 16-21 | ≥21 | (Ozbey *et al.,* 2012) |
| Tetracycline | TE | >30 | - | ≥30 | (Ozbey *et al.,* 2012) |
| Levofloxacin | LEV | >26 | - | ≥26 | (yu *et al.,* 2012) |
| Ciprofloxacin | CIP | >15 | - | ≥15 | (Tanih *et al.,* 2010) |
| Erythromycin | E | >23 | - | ≥23 | (Loivukene *et al.,* 2002) |
| Furazolidone | FX | >13 | 13-21 | ≥21 | (Ogata *et al.,* 2014) |
| Gentamicin | GM | >15 | - | ≥15 | (Tanih *et al.,* 2010) |
| Rifampicin | RD | >21 |  | ≥21 | (Smith, *et al.,* 2014) |

- 1. Powders used for antimicrobial susceptibility testing by minimum inhibitory concentration (MICs) method

| Antimicrobial agent | Source |
| --- | --- |
| Clarithromycin | Abbott laboratories, Argentina |
| Metrnidazole | (Sigma Chemical Co., , USA |
| Tetracycline | (Sigma Chemical Co., , USA |
| Amoxicillin | (Sigma Chemical Co., , USA |
| Rifampicin | Sanofi avents, USA |
| Levofloxacin | Sanofi avents, USA |
| Furazolidone | EPICO, USA |
| Gentamicin | EPICO, USA |
| Ciprofloxacin | EPICO, USA |
| Erythromycin | EPICO, USA |

## Patient data sheet.

Each patient was subjected to the following questionnaire before undergoing upper (GIT) endoscopy:

All personal information will be confidential.

**Thanks for your corporation**

**1-Isolate number: 2-Patient number:**

**3-Isolate type: 4-Patient age (Years only):**

**5-Patient gender (M/F): 6-Patient weight (Kg):**

**7-Do you drink coffee? (Y/N)**

**8-How many cups per day?**

**9-Do you smoke? (Y/N)**

**10-What is your education level?**

**11-Do you take medicines? (including drugs)**

**12-Indication for endoscopy:**

**A-Dyspepsia:**

**B-Heart burn:**

**C-Anemia:**

**D-Persistent vomiting:**

**E-Upper GIT bleeding:**

**13-Results of endoscopy**

**A-Duodenal ulcer**

**B-Gastric ulcer**

**C-Duodenitis**

**D-Gastritis**

**E-Eosophagitis**
