# Supplementary material for: PCR-based RFLP and ERIC-PCR patterns of Helicobacter pylori strains linked to multidrug resistance in Egypt
Source: Sci Rep. 2024 Sep 27;14:22273. doi: 10.1038/s41598-024-72289-z (PMC11436738; doi:10.1038/s41598-024-72289-z)
Supplement: Supplementary file 4 — Supplementary Information 4. [file 41598_2024_72289_MOESM4_ESM.docx]

**APPENDIX D**

**1. Biochemical reactions for identification of *H. pylori*.**

There are many dependable and sensitive biochemical reactions for identification of H. pylori both in biopsy specimen and culture.

**1.1. Ultra rapid urease test (URUT) (Ogata *et al.,* 2002):**

This test is highly specific test for rapid detection of urease enzyme production by *H. pylori* in gastric biopsy specimen.

**1.1.1. Principle**

This test is based on the high concentration of pre-formed urease enzyme in *H. pylori* infected gastric biopsy samples. This will bring about a pH change when placed in a urea containing medium. The sensitivity of the test depends on the number of bacteria present in the sample, which may have consequences for its use when evaluating treatment failures etc. The specificity is very good when the test is read within 1 minute, but declines with the length of the incubation**.** Positive result was indicated by change in the color of the solution from orange to pink within the first minute.

**1.2. CLO (Uotani *et al.,* 2015)**:

Rapid CLO test is a commercial kit used for rapid detection of *H.* *pylori* in gastric biopsy specimen. This test has the same principle as URUT. The test performed by embedding gastric biopsy within agar where pink color was given within 5 minutes in case of positive specimens.

**1.3. Urease agar test (Forbes *et al.,* 2007)**:

This test was used to detect bacterial capacity to analyze urea and production of ammonia and carbon dioxide which shift the pH of the phenol red indicator from neutral (yellow color) to alkaline (pink color). Christensen's urea agar slant was inoculated by streaking with the sterile loop with tested bacteria and incubated at 37ºC for 24 h, pink color indicates positive result while yellow color indicates negative result. *H.* *pylori* is strongly urease producing bacteria.

**1.4. Oxidase test (Washington *et al.,* 2006)**:

This test was employed for the detection of bacterial ability to produce oxidase enzymes. A colony of the test organism was added and rubbed on oxidase disc. Positive result was reported by appearance of blue purple color within 10 seconds while the color of the colony remains the same was a negative result.  *H. pylori* secrete cytochrome oxidase enzyme.

**1.5. Catalase test (Hemraj *et al*., 2013)**:

An amount of pure growth was transferred by a wooden stick to a microscope slide, a drop of 3 % hydrogen peroxide was added on the colony. This test was used to detect the ability of the tested bacteria to produce catalase enzyme. Appearance of bubbles was taken as catalase positive. *H. pylori* is produce catalase enzyme.

**1.5. Nitrate reduction test (Colle & Marr, 1996)**:

This test was determined the ability of the bacteria to reduce nitrate (NO_3_) to nitrite (NO_2_) by the action of nitrate reductase enzyme. The nitrate broth was inoculated and incubated for 96 h, and then 0.1ml of the test reagent was added.

The red color developed within a few min indicate the presence of nitrite and hence the ability of the organism to reduce nitrate. *H. pylori* unable to synthesize nitrate reductase enzyme.

**1.6. H_2_S production test using triple sugar iron (TSI) agar (MacFaddin, 1980)**:

This test was used to determine the bacterial ability to attack a specific carbohydrate incorporated in a basal growth medium, with or without production of gas, along with the determination of possible hydrogen sulfide (H_2_S) production. Using an inoculating loop, the butt of the TSI slant was stabbed to within 1/4 inch from bottom, draw out and fishtail over slant, incubated at 35ºC for 24 h.

**Interpretation:**

Carbohydrate utilization:

1. Fermentation of glucose only:

a. Slant: red colour (alkaline reaction).

b. Butt: yellow colour (acid reaction).

2. Fermentation of glucose and sucrose and/or lactose:

a. Slant: yellow colour (acid reaction).

b. Butt: yellow colour (acid reaction).

3. Neither glucose nor lactose nor sucrose fermented:

a. Slant: red colour (alkaline reaction).

b. Butt: (i) Aerobic organism: no growth (no colour change).

(ii) Facultative organism: red colour (alkaline reaction)

Gas production:

1. Aerogenic: gas production (CO_2_ and H_2_) evident by bubbles in the medium, splitting of medium, complete displacement of the medium from bottom of the tube leaving a clear area or slight indentation of medium from the side of the tube.

2. Anaerogenic: no gas production.

H_2_S production:

The presence of a black precipitate (ferrous sulfide) is evident by a black colour spread throughout the entire butt masking the acidity, a black ring near the top of the butt area or a black precipitate scattered throughout the butt but not entirely masking the acidity present.

*H. pylori* does not ferment sugars neither produce hydrogen sulphide (H_2_S), or gas.

**1.7. Hippurate hydrolysis test (Harvy, 1980)**:

Hippurate hydrolysis test is used to detect the ability of bacteria to hydrolyse hippurate into glycine and benzoic acid by action of hippuricase enzyme present in bacteria. Benzoic acid can be detected rapidly within 2 hours using an oxidizing agent ninhydrin as an indicator. Ninhydrin reacts with glycine to form a deep blue

5 drops of sterile distilled water added to a test tube, using heavy inoculums from fresh culture to make a heavy suspension, using sterile forceps, to place a rapid hippurate disk in the mixture, cap and incubate the tube for two hours at 37 °C. After the two hour incubation period, add 5 drops of the ninhydrin reagent to the mixture. Reincubate at 35-37 °C for -30 minutes. The color change will usually appear in 15 minutes. *H. pylori* is unable to form hippuricase enzyme.

**1.8. Indole test (Hemraj *et al*., 2013; MacFaddin, 1980)**:

Indole test used to detect the bacterial capacity to produce tryptophanase enzyme that hydrolyze tryptophane producing indole, pyruvic acid and ammonia. The presence of indole can be detected by the addition of Kovac’s reagent which reacts with the indole producing a bright red color on the surface of the medium.

Pure bacterial culture was grown in sterile tryptophan or peptone broth for 24 hours before performing the test. Following incubation, add 5 drops of Kovakc’s reagent (isoamyl alcohol, para Dimethylaminobenzaldehyde, concentrated hydrochloric acid) to the culture broth. A positive indole test is indicated by the formation of a red color in the reagent layer on top of the agar deep within seconds of adding the reagent. If a culture is indole negative, the reagent layer will remain yellow or be slightly cloudy**.** *H. pylori* can't form tryptophanase enzyme.

**1.9. Growth with 3.5% NaCl (Jiang and Doyle 1998):**

The organism grew in brain heart infusion broth supplemented with 7% sheep serum and (Dent.) selective supplement, containing 3.5% NaCl under optimum growth condition. There is no growth after 7 days of incubation. The optimal NaCl concentration for growth of *H. pylori* was 0.5 - 1.0%; while concentration of 2.0% NaCl inhibited the growth.

**1.10. Growth with 1% glycine (Minami *et al*., 2004)**.

Glycine is the simplest amino acid and is used as a metabolic product in some bacteria. However, an excess of glycine inhibits the growth of many bacteria. The effect of glycine on *Helicobacter pylori* is not precisely known **(Minami *et al*., 2004)**. The organism grew in brain heart infusion broth supplemented with 7% horse serum and antibiotics, containing 1% glycine under optimum growth condition. There is no growth after 7 days of incubation

**1.11. Growth at varying temperatures (25°C and 42 °C) (Al-Sulami *et al*., 2010)**.

 The organism was inoculated in brain heart infusion broth supplemented with 7% horse serum and antibiotics under optimum growth conditions at 25**°C** and at 42 **°C** .there is no growth for 7 days incubation.

**1.12. Growth on peptone-starch dextrose agar (Al-Sulami *et al*., 2010)**:

*H. pylori* isolates can grow well on laboratory prepared peptone-starch dextrose agar **(Difco)**, producing yellow colonies.

**1.13. Susceptibility Test for Nalidixic Acid and Cephalothin (Hussein 2007)**:

Isolates were inoculated in sterile brain heart infusion broth then incubated at 37°C for 48 hr then, 1 loopfull of this inoculum was spareded on Muller-Hinton agar supplemented with 5% horse blood and then with a sterile forceps, cephalothin and nalidixic acid with disk strength (30 μg) for each one, were placed on the surface of inoculated plate and incubated at 37°C for 24hr under microaerophilic conditions.
