# Supplementary material for: PCR-based RFLP and ERIC-PCR patterns of Helicobacter pylori strains linked to multidrug resistance in Egypt
Source: Sci Rep. 2024 Sep 27;14:22273. doi: 10.1038/s41598-024-72289-z (PMC11436738; doi:10.1038/s41598-024-72289-z)
Supplement: Supplementary file 5 — Supplementary Information 5. [file 41598_2024_72289_MOESM5_ESM.docx]

***PCR-based RFLP and ERIC-PCR* patterns of *Helicobacter pylori* strains linked to multidrug resistance in Egypt**

**Supplementary data**

Whole bacterial genome was extracted from the 50 *H. pylori* isolates and detected using 2.0 % agarose. DNA then visualized by placing on a UV light source and photographed directly.


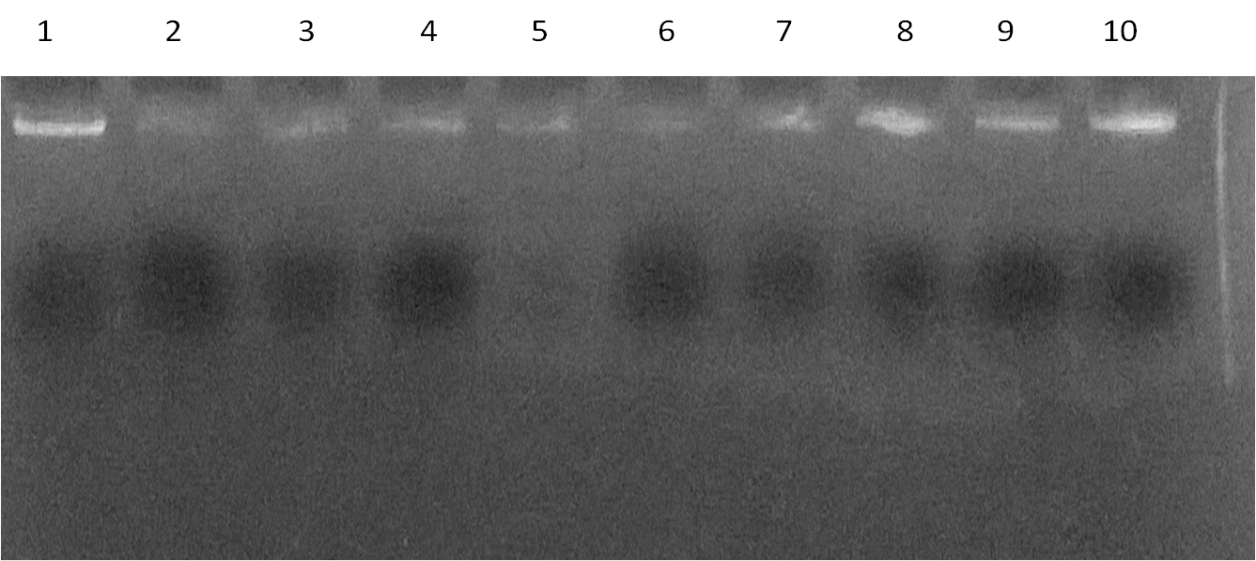


**Agarose gel electrophoresis of *H. pylori* DNA. Lane 1-10: genomic DNA of different *H. pylori* isolates.**

1. **RFLP-PCR**


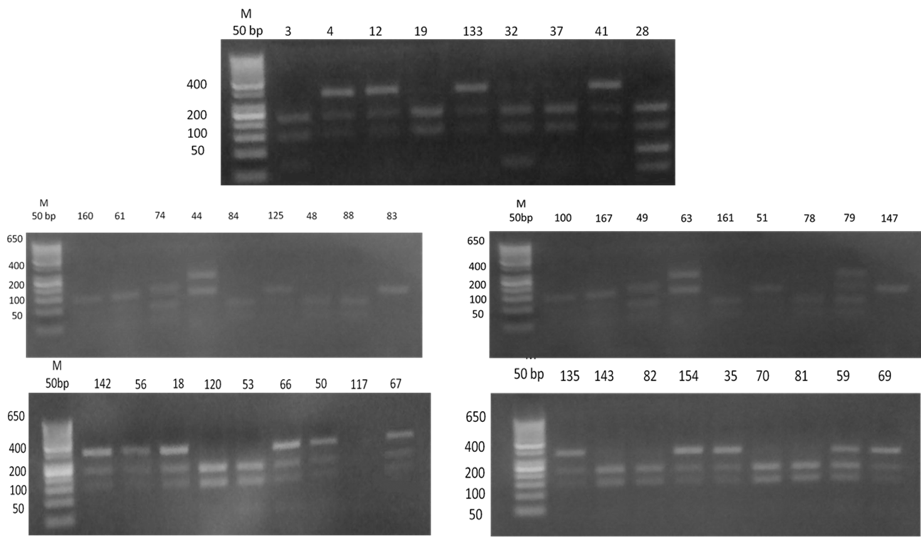


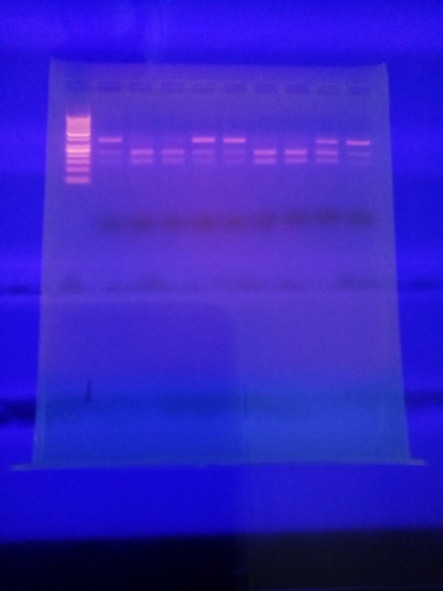


Figure 3: PCR-based RFLP patterns of *H. pylori* isolates digested with *Mbo*I restriction enzyme, (n=44). Restriction enzyme analysis of the 500 bp yielded 11 bands with different sizes indicative of 15 genotypes and are denoted in sky blue boxes. Of the fifteen genotypes, the most predominant genotype were M4, followed by M9, and then M2 as shown in the following table. The original gel is identical to the section of the figure marked with skyblue rectangle and representing the bottom right-hand side of the four sections. The four sections present the 44 isolates.

| **Genotypes** | **DNA bp** | **Isolates**  **No. (%)** | **Genotypes** | **DNA bp** | **Isolates**  **No. (%)** |
| --- | --- | --- | --- | --- | --- |
| **M1** | 100,175,275 | 1(2.3) | **M9** | 150,275 | 7(15.9) |
| **M2** | 75,125,200 | 6(13.6) | **M10** | 30,50,100,150 | 1(2.3) |
| **M3** | 50,125,200 | 3(6.8) | **M11** | 50,140,250 | 1(2.3) |
| **M4** | 100,150,250 | 8(18.1) | **M12** | 35,50,225 | 3(6.8) |
| **M5** | 125,350 | 3(6.8) | **M13** | 75,200,275 | 1(2.3) |
| **M6** | 75,150,300 | 4(9.1) | **M14** | 25,50,350 | 2(4.5) |
| **M7** | 35,100,200 | 1(2.3) | **M15** | 75,175,225 | 2(4.5) |
| **M8** | 25,100,200 | 1(2.3) | **-** | - | - |

1.
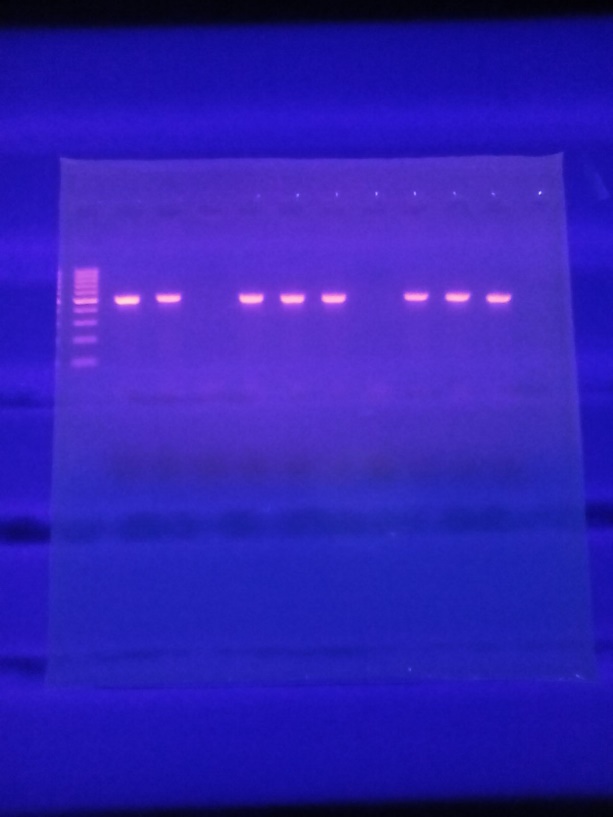
**PCR amplification products of *H-pylori* ureC gene**

Figure 2: PCR amplification products of *H-pylori ureC* gene yielded 500 bp bands. V, negative control and M, 100BP molecular weight marker. The original gel is identical to figure 2.

1. **ERIC-PCR:**


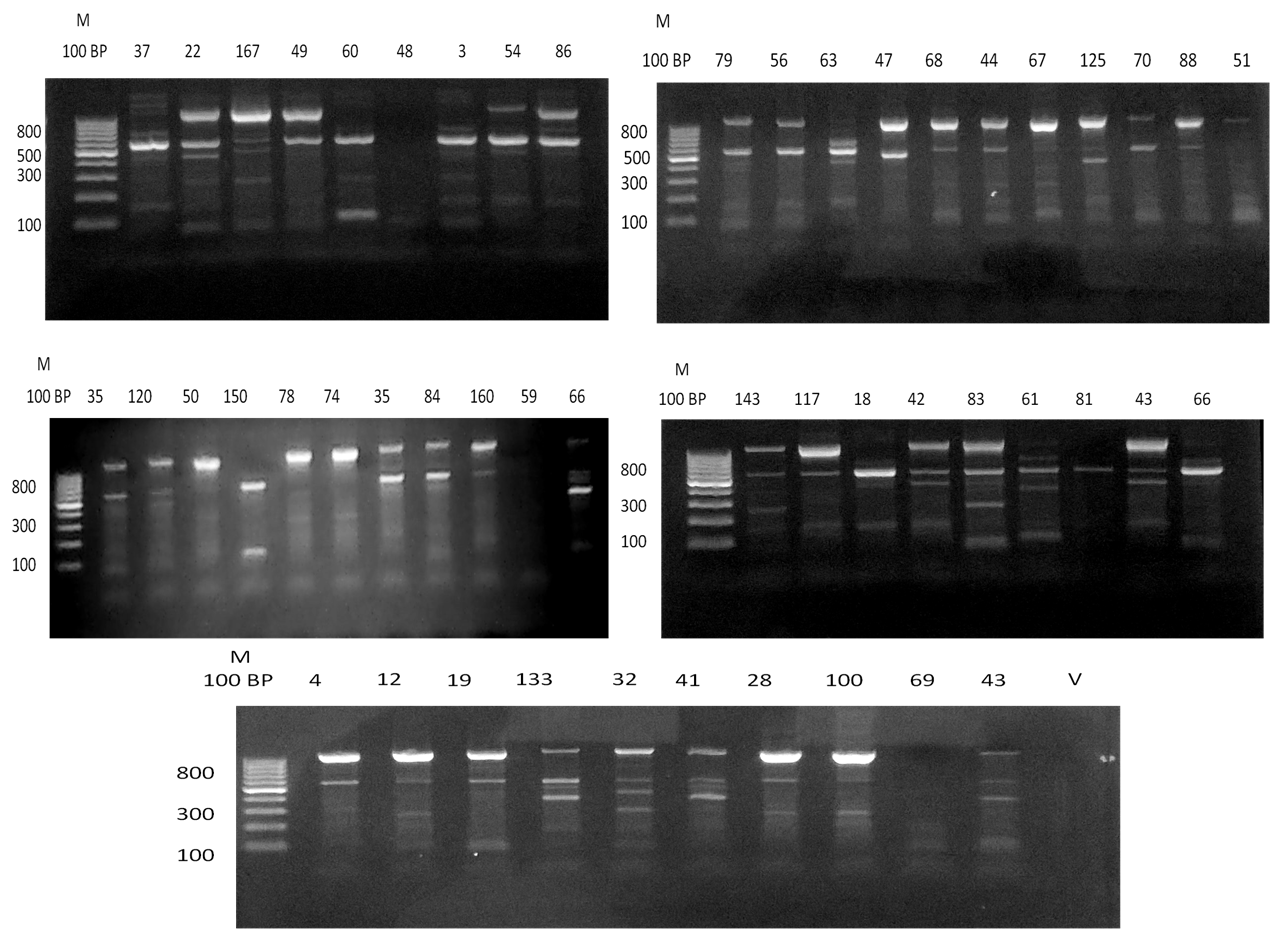


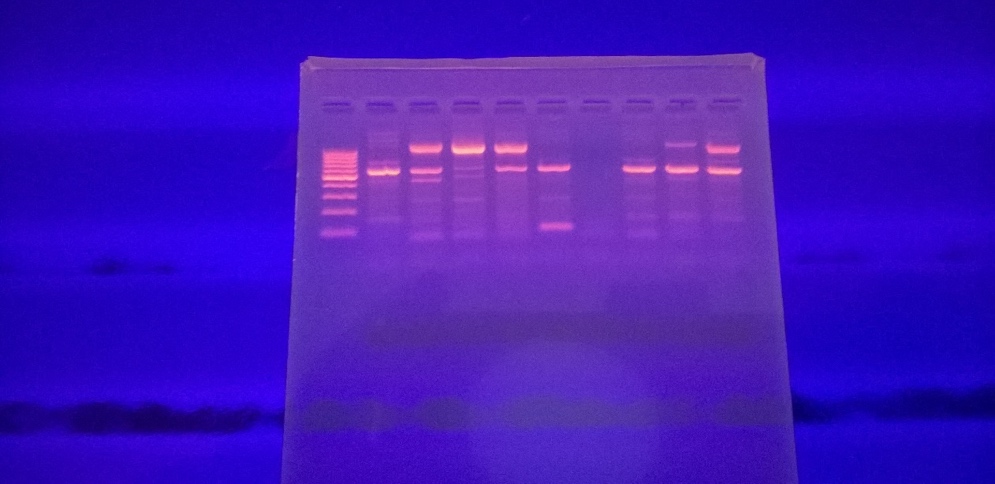
This gel is identical with the first section on the upper left-hand side of figure 1.


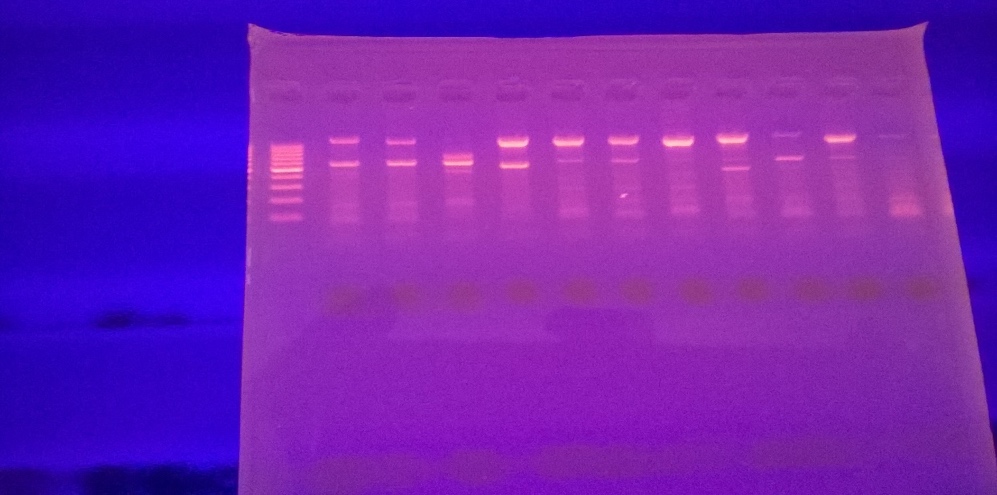
This gel is identical with the section on the upper right-hand side of figure 1.


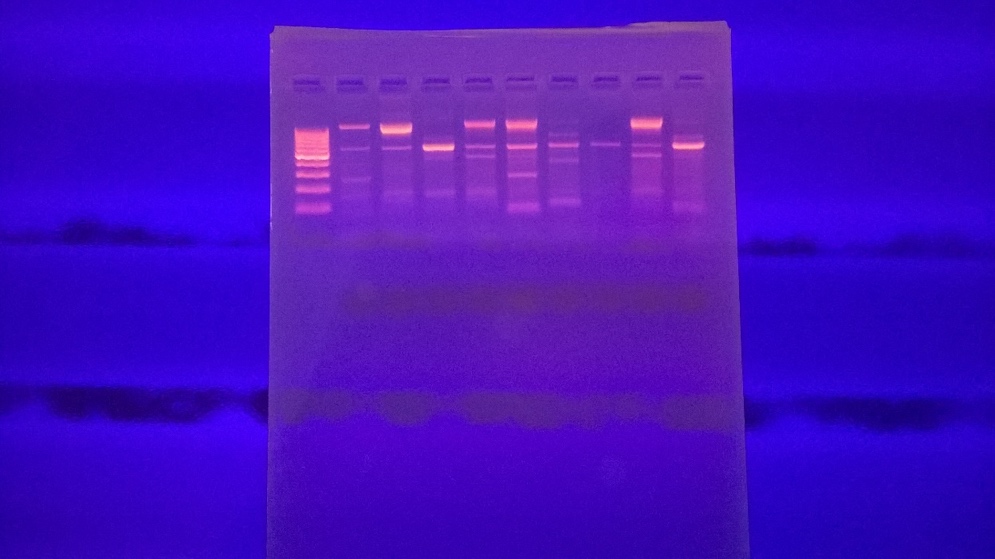
This gel is identical with the section on the middle right-hand side of figure 1.


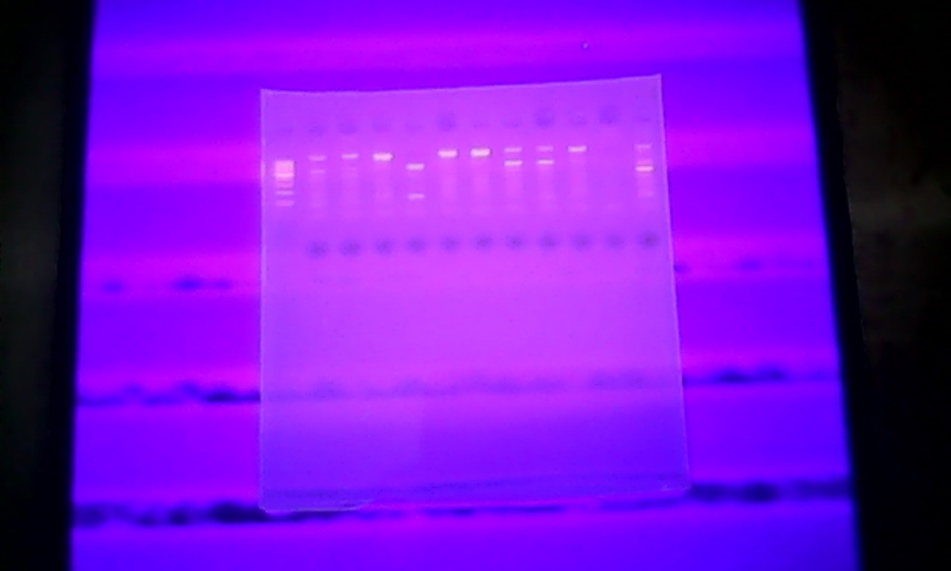
This gel is identical with the section on the middle left-hand side of figure 1.


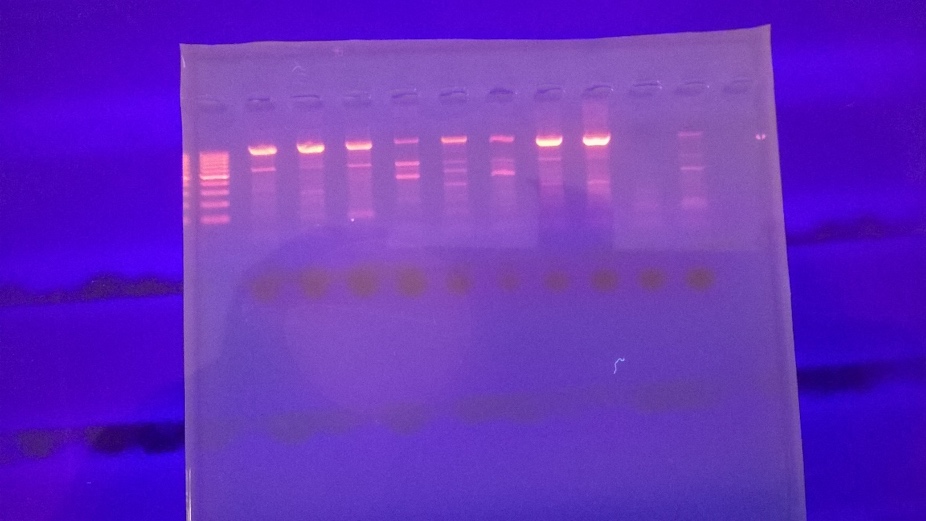
This gel is identical with the section on the bottom of figure 1.

Figure 1: DNA fingerprints generated by ERIC-PCR analysis of *H. pylori* isolated products on 2% agarose gel: M is 100 bp DNA ladder, (V) is a negative control, (n=50). We validated phenotyping data by running ERIC PCR fragments. The red boxes denote typical 126bp fragment from all isolates. The blue-sky boxes denote nine other bands different in sizes resulted in 22 genotypes. This polymorphic pattern indicates that the ERIC sequences were dispersed in the *H pylori* chromosome at different locations separated by distances. Of the 22 genotypes, the most predominant genotype was G2, followed by G4, as presented in the following table.

| **Genotype** | **DNA bp** | **Isolates**  **No. (%)** | **Genotype** | **DNA bp** | **Isolates**  **No. (%)** |
| --- | --- | --- | --- | --- | --- |
| **G1** | 280,600,750 | 2(4) | **G12** | 500,700,>1000 | 4(8) |
| **G2** | 600,>1000 | 8(16) | **G13** | 500,600,750,>1000 | 1(2) |
| **G3** | 700 | 2(4) | **G14** | 550,>1000 | 1(2) |
| **G4** | 280,600,>1000 | 7(14) | **G15** | 300,500,>1000 | 1(2) |
| **G5** | 400,500,600,>1000 | 2(4) | **G16** | 500,650,>1000 | 2(4) |
| **G6** | 280,450,600,>1000 | 2(4) | **G17** | 400,500,600,800 | 1(2) |
| **G7** | 500,600,800 | 2(4) | **G18** | 600,750 | 2(4) |
| **G8** | 400,450,600,>1000 | 2(4) | **G19** | 400,500,600,800,>1000 | 1(2) |
| **G9** | 400,500,>1000 | 2(4) | **G20** | 350,550,>1000 | 1(2) |
| **G10** | 280,400,600,850 | 2(4) | **G21** | 200,650,>1000 | 1(2) |
| **G11** | 150 | 3(6) | **G22** | 280,400,650,>1000 | 1(2) |

**Materials**

***1. Chemicals, biological materials and standard solutions.***

1.1. Chemicals:

Glycerol, ethanol 70 %, conc. HCl, disodium hydrogen phosphate (Na2 HPO4), potassium acetate, potassium hydroxide (KOH), sodium chloride (NaCl), potassium nitrate (KNO3 nitrite free), potassium iodide, iodine crystals, methanol, barium chloride dihydrate (BaCl2.2H2O), 1 % sulfuric acid, hydrogen peroxide 3 % and urea were supplied from El Nasr pharmaceutical chemicals Co., Cairo, Egypt.

Phenol red, isoamyl alcohol peptone, potassium chloride, tris base, tris-HCL, sodium acetate.3H2O, boric acid, bromophenol blue, sucrose, ethidium bromide and acetic acid were supplied from Sigma Aldrich Chemical Co. LTD, Germany.

Crystal violet, saffranin, and Leoffler & apos, methylene blue were supplied from Nice chemicals. Pvt. Ltd. India. Electrophoresis-grade agarose powder, tetramethylethylenediamine (TEMED) were supplied from GIBCO Bethesda Research Lab., U.S.A. yeast extract, and agar were obtained from Difco Laboratories.

1.2. Biological materials:

Sheep blood used in this study was obtained from Veterinary Serum and Vaccine Research Institute, Cairo.

1.3. Standard solutions.

1.3.1. Turbidity standard (McFarland 3.0) (Baron & Fienegold, 1990):

Turbidity standard (McFarland No. 0.5) was prepared by adding 3.0 ml of 1.175 % barium chloride dihydrate (BaCl2.2H2O) to 99.5 ml of 1 % sulfuric acid in graduated cylinder, 10 ml of mixture were put in sterile test tubes and stored in the dark at room temperature. The absorbance was measured by a spectrophotometer at a wavelength of 625 nm. The acceptable absorbance range for the standard is 0.08 - 0.13. The contents were mixed well prior to standardizing bacterial cells number which was equivalent to 1.5 × 10 8 CFU/ml.

1.3.2. Normal saline:

This solution was prepared according to Hussein et al. (2013) by dissolving 4.24 g of NaCl in 500 ml of distilled water to obtain 0.145 M NaCl. Thereafter it was autoclaved and stored at 4^o^ C.

**2. Reagents, stains and identification kits:**

Compositions and preparations of reagents and stains are as follows:

2.1. Reagents:

2.1.1. Reagents for nitrate reduction test

2.1.2. Catalase reagent (3 % H2O2 )

2.1.3. Kovacs’ reagent for indole test

2.1.4. Urea (10%) solution for URUT

2.1.5 Ethidium bromide solution (10 mg/ml)

2.1.6. Ninhydrin solution

2.1.7. 1xTEA buffer (pH 8)

2.1.8. 3 M sodium acetate (pH 4.8 and pH 5.7)

2.1.9. Electrophoresis buffer (Tris-borate-EDTA buffer) (TBE)

2.1.10. Agarose gel (0.8 % and 3 %)

2.1.11. API NaCl 0.85 % Medium

2.1.12. API ZYM A and API ZYM B reagents

2.1.13. Phosphate buffer saline (PBS)

2.1.13. MboI Restriction enzyme

2.1.14. Reagents for DNA extraction

**3.2. Stains**

3.2.1. Gram stain

3.2.2. Simple stain with Leoffler&apos;s methylene blue

3.3. Identification kits

3.3.1. Oxidase discs

Oxidase discs were obtained from HIMEDIA Laboratories, PVT. Limited, India.

3.3.2. Hippurate discs

Remel hippurate discs were obtained from Thermo Scientific™, USA.

**4. PCR oligonucleotides used in this study:**

ERIC primers (ERIC-1R and ERIC-R) were used for detection of interspersed ERIC sequences within *H. pylori* genome (Hussien et al., 2004). On the other hand, UreC (GlmM) gene *in H. pylori*, which encodes a phosphoglucoseamine mutase, was amplified using ureC-U and ureC-L are primers used for detection of ureC gene (Navabakbar and Salehi, 2004)

**Antimicrobial susceptibility testing**

**Table SD.1: Powders used for antimicrobial susceptibility testing by minimum inhibitory concentration (MICs) method and the sources where they come from**

| **Antimicrobial** **agent** | **Source** |
| --- | --- |
| **Clarithromycin** | Abbott laboratories, Argentina |
| **Metrnidazole** | (Sigma Chemical Co., , USA |
| **Tetracycline** | (Sigma Chemical Co., , USA |
| **Amoxicillin** | (Sigma Chemical Co., , USA |
| **Rifampicin** | Sanofi avents, USA |
| **Levofloxacin** | Sanofi avents, USA |
| **Furazolidone** | EPICO, USA |
| **Gentamicin** | EPICO, USA |
| **Ciprofloxacin** | EPICO, USA |
| **Erythromycin** | EPICO, USA |

**5. Antimicrobial agents used for antimicrobial susceptibility testing**

**Table SD.2:** **Discs of antimicrobial agents and their potencies used for antimicrobial susceptibility testing of *H. pylori* isolates.**

| **Antimicrobial** **agent** | **Disc** **strength** |
| --- | --- |
| **Amoxicillin** **(AM)** | 10 μg |
| **Clarithromycin** **(CLA)** | 15 μg |
| **Metronidazole** **(MTZ)** | 5 μg |
| **Tetracycline** **(TE)** | 30 μg |
| **Levofloxacin** **(LEV)** | 5 μg |
| **Ciprofloxacin** **(CIP)** | 10 μg |
| **Erythromycin** **(E)** | 15 μg |
| **Furazolidone (FX)** | 100 μg |
| **Gentamicin (GM)** | 10 μg |
| **Rifampicin (RD)** | 5 μg |

**Table SD.3: Zone sizes for each antimicrobial agent used for antimicrobial susceptibility testing of *H. pylori* isolates.**

| Antimicrobial agent | Code | Zone size breakpoints (mm) | | | Reference |
| --- | --- | --- | --- | --- | --- |
|  |  | R | I | S |  |
| Amoxicillin | AM | >25 | - | ≥25 | **(Ozbey *et al.,* 2012)** |
| Clarithromycin | CLA | >30 | - | ≥30 | **(Ozbey *et al.,* 2012)** |
| Metronidazole | MTZ | >16 | 16-21 | ≥21 | **(Ozbey *et al.,* 2012)** |
| Tetracycline | TE | >30 | - | ≥30 | **(Ozbey *et al.,* 2012)** |
| Levofloxacin | LEV | >26 | - | ≥26 | **(yu *et al.,* 2012)** |
| Ciprofloxacin | CIP | >15 | - | ≥15 | **(Tanih *et al.,* 2010)** |
| Erythromycin | E | >23 | - | ≥23 | **(Loivukene *et al.,* 2002)** |
| Furazolidone | FX | >13 | 13-21 | ≥21 | **(Ogata *et al.,* 2014)** |
| Gentamicin | GM | >15 | - | ≥15 | **(Tanih *et al.,* 2010)** |
| Rifampicin | RD | >21 |  | ≥21 | **(Smith, *et al.,* 2014)** |

**6. Tools used in the current study:**

6. All tools used in the current study i.e. Sterile cotton swabs, Ice box, test tubes, capped eppendorf tubes, screw capped wide-mouthed glass tubes, hypodermic needle, slides, Petri-dishes, membrane filter (0.22 μm), flasks, a rubber stopper Erlenmeyer sidearm flask, plastic syringes, anaerobic EZ GasPack campy, measuring cylinders, anaerobic jar, beakers and graduated pipettes were sterile, disposable.

**7. Instruments used in the current study:**

7.1. PCR (Biometra, USA)

7.2. Autoclave. (Hirayama Manufacturing Corporation, Japan)

7.3. Water bath

7.4. Oven

7.5. Endoscopy machine (Olympus Videotrolley tv-z CLE-10 machine, USA)

7.6. Incubator (Heraeus, USA)

7.7. Vortex (Fisher Scientific)

7.8. Horizontal Gel Electrophoresis Apparatus:

A. Gel casting platform (BioRad)

B. Gel combs (Slot forms) (Bio-Rad)

C. DC power supply (GIBCO-BRL)

7.9. UV Ttransluminator (Fotodyne, Hartland, Wi, U.S.A.)
