# Supplementary material for: PCR-based RFLP and ERIC-PCR patterns of Helicobacter pylori strains linked to multidrug resistance in Egypt
Source: Sci Rep. 2024 Sep 27;14:22273. doi: 10.1038/s41598-024-72289-z (PMC11436738; doi:10.1038/s41598-024-72289-z)
Supplement: Supplementary file 6 — Supplementary Information 6. [file 41598_2024_72289_MOESM6_ESM.docx]

***PCR-based RFLP and ERIC-PCR* patterns of *Helicobacter pylori* strains linked to multidrug resistance in Egypt**

**Supplementary tables**

**Supplementary table S.T. 1: Isolation rates of *H. pylori* obtained from different culture media**

| **Culture media** | **Isolation rate** | |
| --- | --- | --- |
|  | **No.** | **%*** |
| **Selective brain heart infusion blood agar (BHI)** | 112/167 | 67 |
| **Selective brucella blood agar (BAA)** | 98/167 | 58.6 |
| **Selective Columbia blood agar (CBA)** | 95/167 | 56.8 |
| **Classic Columbia agar (CCA)** | 74/167 | 44.3 |

***Numbers were calculated as a percentage of the total number of isolates**

**Supplementary table S.T.2: Complaint expressed by patients and their diagnosis using the upper GIT endoscopy**

| Variables | No. | | %* | |
| --- | --- | --- | --- | --- |
| Epigastric pain | 133/167 | | 79.6 | |
| Heart burn | 19/167 | | 11.4 | |
| Dysphagia | 7/167 | | 4.2 | |
| Weight loss | 6/167 | | 3.6 | |
| Vomiting | 54/167 | | 32.3 | |
| Distention | 37/167 | | 22.1 | |
| Constipation | 21/167 | | 12.6 | |
| Diagnosis | Occurrence | | *H. pylori* +ve | |
|  | No. | %* | No. | %** |
| Gastroesophageal reflux disease (GERD) | 32 | 13.8 | 16 | 50.0 |
| Gastric ulcer (GU) | 43 | 18.5 | 32 | 74.4 |
| Duodenal ulcer (DU) | 19 | 8.2 | 11 | 57.9 |
| Duodenitis | 36 | 15.6 | 25 | 69.4 |
| Gastritis | 72 | 31.0 | 64 | 88.8 |
| Normal mucosa | 30 | 12.9 | 19 | 63.3 |

***Numbers were calculated as a percentage of the total number of isolates**

**Supplementary Table S.T.3.: API ZYM cutoff table**.

| NO. | ENZYME ASSAYED FOR | SUBSTRATE | pH | RESULT | |
| --- | --- | --- | --- | --- | --- |
|  |  |  |  | **POSITIVE** | **NEGATIVE** |
| 1 | **Control** |  |  | Colorless or color of the sample if it has an intense coloration | |
| 2 | **Alkaline phosphatase** | 2-naphthyl phosphate | 8.5 | Violet | Colorless Or very pale yellow |
| 3 | **Esterase (C 4)** | 2-naphthyl butyrate | 6.5 | Violet |  |
| 4 | **Esterase Lipase (C 8)** | 2-naphthyl caprylate | 7.5 | Violet |  |
| 5 | **Lipase (C 14)** | 2-naphthyl myristate | 7.5 | Violet |  |
| 6 | **Leucine arylamidase** | L-leucyl-2-naphthylamide | 7.5 | Orange |  |
| 7 | **Valine arylamidase** | L-valyl-2-naphthylamide | 7.5 | Orange |  |
| 8 | **Cystine rylamidase** | L-cystyl-2-aphthylamide | 7.5 | Orange |  |
| 9 | **Trypsin** | N-benzoyl-DL-arginine-2-naphthylamide | 8.5 | Orange |  |
| 10 | **α -chymotrypsin** | N-glutaryl-phenylalanine-2-naphthylamide | 7.5 | Orange |  |
| 11 | **Acid phosphatase** | 2-naphthyl phosphate | 5.4 | violet |  |
| 12 | **Naphthol-AS-BI-phosphohydrolase** | Naphthol-AS-BI-phosphate | 5.4 | blue |  |
| 13 | **α -galactosidase** | 6-Br-2-naphthyl-􀁄D-galactopyranoside | 5.4 | violet |  |
| 14 | **ß-galactosidase** | 2-naphthyl-ßD-galactopyranoside | 5.4 | violet |  |
| 15 | **ß-glucuronidase** | Naphthol-AS-BI-ßD-glucuronide | 5.4 | blue |  |
| 16 | **α –glucosidase** | 2-naphthyl- α D-glucopyranoside | 5.4 | violet |  |
| 17 | **ß-glucosidase** | 6-Br-2-naphthyl-ßD-glucopyranoside | 5.4 | violet |  |
| 18 | **N-acetyl-ß-glucosaminidase** | 1-naphthyl-N-acetyl-ßD-glucosaminide | 5.4 | brown |  |
| 19 | **α -mannosidase** | 6-Br-2-naphthyl-α D-mannopyranoside | 5.4 | violet |  |
| 20 | **α –fucosidase** | 2-naphthyl- α L- fucopyranoside | 5.4 | violet |  |

**Supplementary table S.T.4: Genotyping of *H. pylori* strains by ERIC-PCR**

| **Genotype** | **Total** | | **Genotype** | **Total** | |
| --- | --- | --- | --- | --- | --- |
|  | **No.** | **%*** |  | **No.** | **%*** |
| **G_1_** | 2 | 4 | **G_12_** | 4 | 8 |
| **G_2_** | 8 | 16 | **G_13_** | 1 | 2 |
| **G_3_** | 2 | 4 | **G_14_** | 1 | 2 |
| **G_4_** | 7 | 14 | **G_15_** | 1 | 2 |
| **G_5_** | 2 | 4 | **G_16_** | 2 | 4 |
| **G_6_** | 2 | 4 | **G_17_** | 1 | 2 |
| **G_7_** | 2 | 4 | **G_18_** | 2 | 4 |
| **G_8_** | 2 | 4 | **G_19_** | 1 | 2 |
| **G_9_** | 2 | 4 | **G_20_** | 1 | 2 |
| **G_10_** | 2 | 4 | **G_21_** | 1 | 2 |
| **G_11_** | 3 | 6 | **G_22_** | 1 | 2 |

*Numbers were calculated as a percentage of the total number of isolates

**Supplementary table S.T.5: Genotyping of *H. pylori* strains by PCR-RFLP technique (n=44)**

| **Genotype** | **Total** | | **Genotype** | **Total** | |
| --- | --- | --- | --- | --- | --- |
|  | **No.** | **%*** |  | **No.** | **%*** |
| M_1_ | 1 | 2.3 | M_9_ | 7 | 15.9 |
| M_2_ | 6 | 13.6 | M_10_ | 1 | 2.3 |
| M_3_ | 3 | 6.8 | M_11_ | 1 | 2.3 |
| M_4_ | 8 | 18.1 | M_12_ | 3 | 6.8 |
| M_5_ | 3 | 6.8 | M_13_ | 1 | 2.3 |
| M_6_ | 4 | 9.1 | M_14_ | 2 | 4.5 |
| M_7_ | 1 | 2.3 | M_15_ | 2 | 4.5 |
| M_8_ | 1 | 2.3 | - | - | - |

***Numbers were calculated as a percentage of the total number of isolates**

**Supplementary table S.T.6: Range of antibiotic concentrations used in MIC method.**

| Antimicrobial agents | Code | Range of antibiotics concentrations (mg/L) |
| --- | --- | --- |
| Amoxicillin | AM | 0.125-64 |
| Clarithromycin | CLA | 0.125-16 |
| Metronidazole | MTZ | 0.5-256 |
| Tetracycline | TE | 0.125-8 |
| Levofloxacin | LEV | 0.125-8 |
| Ciprofloxacin | CIP | 0.004-0.5 |
| Rifampicin | RD | 0.125-8 |
| Furazolidone | FX | 0.015-64 |
| Gentamicin | GM | 0.5-32 |
| Erythromycin | E | 0.125-8 |

**Supplementary table S.T.7: The MIC breakpoints of antimicrobial agents according to The EUCAST protocol (2014).**

| Antimicrobial agents | MICs breakpoint values (mg/L) | | |
| --- | --- | --- | --- |
|  | **S** | **I** | **R** |
| Amoxicillin | ≤ 0.25 | 0.25-0.5 | > 0.5 |
| Clarithromycin | ≤ 0.25 | 0.25-0.5 | > 0.5 |
| Metronidazole | ≤ 8 | - | > 8 |
| Tetracycline | ≤ 1 | - | > 1 |
| Levofloxacin | ≤ 1 | - | > 1 |
| Ciprofloxacin | ≤ 1 | - | > 1 |
| Rifampicin | ≤ 1 | - | > 1 |
| Furazolidone | ≤ 4 | - | >4 |
| Gentamicin | ≤ 16 | - | >16 |
| Erythromycin | ≤ 2 | - | >2 |

**MIC: minimum inhibitory concentration, R: resistant, S: susceptible, I: intermediate susceptible.**

**Supplementary table S.T.8. (A): Primers’ design used for ERIC-PCR genotyping**

| **Primer** | **Sequence** |
| --- | --- |
| **(Foreword): ERIC-1R** | 5′ ATGTAAGCTCCTGGGGATTCAC 3′ |
| **(Reverse): ERIC-2R** | 5′ AAGTAAGTGACTGGGGTGAGCG 3′ |

**Supplementary table S.8 (B): Primers’ design used for PCR-RFLP genotyping**

| **Primer** | **Sequence** |
| --- | --- |
| **(Foreword): *ure*C-U** | 5′-CCC TCA GAC CAG TCC CAA AAA-3′ |
| **(Reverse): *ure*C-L** | 5′-AAG AAG TCA AAA ACG CCC CAA AAC-3′ |

**Supplementary table S.T.9 A: Components of the reaction mixture for PCR-RFLP.**

| Component | Volume |
| --- | --- |
| 2x PCR master mix solution (i-TaqTM) | **25.0 𝜇l** |
| Template DNA | **5.0 𝜇l** |
| Primer (F) | **10 PM** |
| Primer (R) | **10 PM** |
| Distilled water | **18 𝜇l** |
| Total volume | **50 μl** |

**Supplementary table S.T. 9 B: Components of 2x PCR master mix solution (i-TaqTM) used in the present study.**

| Component | Concentration |
| --- | --- |
| i-Taq TM DNA polymerase (5 U/ μl) | **2.5 U** |
| DNTPs | **2.5 mM each** |
| PCR reaction buffer | **1x** |
| Gel loading buffer | **1x** |
